# Analysis of Poly-3-Hydroxybutyrate Production with Different Microorganisms Using the Dynamic Simulations for Evaluation of Economic Potential Approach

**DOI:** 10.1021/acsomega.4c11178

**Published:** 2025-06-11

**Authors:** Willians de Oliveira Santos, Rafael David de Oliveira, José Gregório Cabrera Gomez, Galo Antonio Carrillo Le Roux

**Affiliations:** † Department of Chemical Engineering Polytechnic School, University of São Paulo, Av. Prof. Lineu Prestes, 580, São Paulo 05508-220, Brazil; ‡ Department of Chemical Engineering, 8018 Norwegian University of Science and Technology (NTNU), Torgarden, NO-7491, Trondheim 8900, Norway; § Institute of Biomedical Sciences, Bioproducts Laboratory, University of São Paulo, Av. Prof. Lineu Prestes, 2415, São Paulo 05508-000, Brazil

## Abstract

Concerns with sustainability have led to increasing interest in bioprocesses in the last decades. In particular, environmental problems with plastic disposal have been a major issue. Bioplastics such as poly-3-hydroxybutyrate (PHB) are potential substitutes since they are biodegradable and less toxic. However, their production costs are high and optimization is required. Many works have therefore aimed to build strains capable of higher product yields. But in the case of products that share a precursor with biomass and in the case of intracellular metabolites, a trade-off may occur. Not only yield but also final biomass, titer, and productivity have to be considered. Therefore, this work presents an approach named Dynamic Simulations for Evaluation of Economic Potential (DySEEP), which uses dynamic flux balance analysis (DFBA) and an economic metric as a function of the bioprocess parameters for evaluation of the production of bioproducts of industrial interest. As a case study, the PHB production potential of recombinant *Cupriavidus necator*, *Escherichia coli*, and *Saccharomyces cerevisiae* was analyzed. While some key polymer properties cannot be predicted due to the nature of DFBA simulations, the PHB production is a good case study to highlight the trade-off between biomass and product formation. It was identified that for growth-associated production with recombinant *E. coli* and the NADPH-dependent PHB synthesis pathway, the scenario that starts positive cash flows is when a yield of 0.37 g/g and its respective final biomass, titer, and productivity is achieved, and the maximum theoretical profit would be achieved when a yield of 0.50 g/g and its respective parameters is reached. Furthermore, comparison between the internal flux distribution of the best scenario identified and the flux distributions of a simulation constrained with experimental data from the literature allowed the suggestion of genetic modifications that could enhance PHB production, such as knockouts for interruption of the oxidative phase of the pentose phosphate pathway, of the acetate production reaction, and of the reaction catalyzed by the 2-oxoglutarate dehydrogenase enzyme or downregulation of the TCA cycle, setting therefore potential targets for metabolic engineering strategies. For nongrowth-associated production with both recombinant *E. coli* and *C. necator*, the scenario at which cash flow starts to become positive is when 40% (mol) of the available glucose is used in the growth phase and the remaining 60% is used in the production phase, and the scenario that leads to the maximum theoretical profit, within a realistic maximum PHB content, is when 20% (mol) is used in the growth phase and the remaining 80% in the production phase, information that sets targets for bioreactor operation strategies such as defining the moments for nutrient limitation and for synthetic biology by showing when to activate genetic toggle-switches.

## Graphical abstract

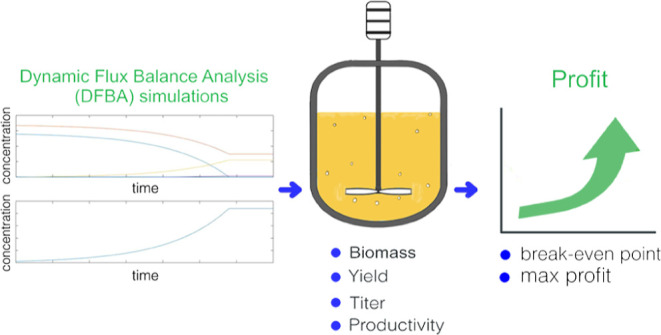

## Introduction

The increasing awareness of industrial processes’ environmental impacts has been driving the search for sustainable processes in the last decades.^1,2^ A shift from nonrenewable petroleum-based industries to processes based on renewable resources has been pursued, and with that, the bioprocess industry has seen great developments over the years.^2,3^ Still, there are many instances where the economic viability becomes a problem.^4,5^ While the petroleum industry has well-established technology and high production at relatively low costs, some bioprocesses still require optimization in order to be economically competitive, such as the production of bioplastics.^1,5–7^

Metabolic engineering has been used to build strains that are capable of using cheaper carbon sources and that can reach higher product yields, improving the economic viability of many potential bioprocesses.^1,3,8^ Computational tools and mathematical approaches are used to obtain useful information about microorganisms and to assist in the identification of genetic modifications that can improve the production of desired products.^9–11^ Databases like the Kyoto Encyclopedia of Genes and Genome (KEGG) with annotated genomic data for several different bacteria became easily accessible, which allowed the construction of automated or manually curated models.^12^ For instance, the Systems Biology Research Group from the University of California built the BiGG Models, a repository containing several metabolic network reconstructions,^12^ and there are other important genome-scale metabolic model (GSMM) repositories such as MetaNetX, BioModels, Virtual Metabolic Human, and MEMOSys.^12–14^

With the metabolic network reconstruction of a microorganism, a few mathematical methods can be used to compute a possible set of internal metabolic fluxes and to predict some phenotypical characteristics under certain conditions.^11^ One such method, which uses stoichiometric models with constraints, is Flux Balance Analysis (FBA). FBA finds a metabolic flux distribution that maximizes or minimizes an expression deemed as an objective function. The objective function is often something that describes the cell’s behavior, such as biomass formation. The method assumes some simplification hypotheses such as steady state, and it also does not account for gene regulation or reaction kinetics and instead relies on reaction stoichiometry and constraints based on thermodynamic feasibility and experimental data whenever available. Nevertheless, FBA allows the computation of important biological information such as ATP and cofactor generation or demand, maximum growth rate given a carbon source uptake rate, essentiality of genes, effect of knockouts, and maximum product yields.^11,15–18^ Finding the theoretical maximum yield using FBA is useful because, even if it may not be quite achievable in practice, it indicates the upper limit for what can be achieved, allowing the pre-evaluation of potential bioprocesses to some degree and assisting in metabolic engineering strategies that aim to create modified strains capable of higher yields.^15–17^

But while improving yield can be beneficial, there are other important bioprocess parameters such as final biomass concentration, titer, and productivity that have to be taken into consideration during the design process.^19^ For example, in the case of growth-associated products or intracellular metabolites, a higher yield may come at the cost of less biomass formation and longer operation times, which in turn can negatively affect the productivity.^19,20^ Higher titers may decrease downstream separation costs, but if it takes too long to reach these high product concentrations, it may not be worth it. The best option may not necessarily be the highest possible yield, or titer, or productivity but instead a set of these three parameters that, when upstream, bioreactor operation, and downstream costs are considered, actually lead to the best performance.^19^

After the introduction of FBA, several other mathematical approaches building upon it were introduced with the intention of amending some of its shortcomings. Dynamic flux balance analysis (DFBA) uses kinetic expressions to describe nutrient, oxygen, and carbon source uptakes, FBA to describe the internal metabolism, and mass balance equations for the external metabolites.^21,22^ This allows the determination of other important bioprocess parameters such as product titer and productivity.^21,22^ Therefore, DFBA can be used to evaluate the performance of simulations that explore the trade-off between biomass and product formation when applying a proper evaluation metric that is a function of the biomass, yield, titer, and productivity such as the monthly gross profit. By calculating the monthly gross profit from simulations that fully explore the trade-off between biomass and product formation, one can properly rank these simulations in terms of their performance in a bioprocess and identify the scenarios that would lead to positive cash flows and the maximum theoretical production potential of the bacteria being used in the simulations. In addition, while approaches such as constraint-based optimization may not perfectly describe the behavior of cells in vivo due to simplifications such as not accounting for metabolic regulations, the identified in silico best metabolic flux distributions can then be seen as potential targets to guide strategies ranging from bioreactor operation optimization to genetic modifications of real strains.^15–17^ Expanding upon the literature of DFBA, this work presents an approach that uses DFBA together with a suitable economic metric, named Dynamic Simulations for Evaluation of Economic Potential (DySEEP), for the evaluation of the production potential of bioproducts of industrial interest. The DySEEP approach identified potential targets for future metabolic engineering and synthetic biology efforts.

As a case study, the production of poly-3-hydroxybutyrate was analyzed using the DySEEP approach. Polyhydroxyalkanoates (PHA) are a class of thermoplastic polyesters used for bioplastic production.^23^ Poly-3-hydroxybutyrate (PHB) is the most well-known PHA, but there are many other polymers and even copolymers such as P3HB-*co*-3HV and P3HB-*co*-3HHx, each with their own thermomechanical properties, greatly expanding the spectrum of PHA applications.^24^ PHAs such as PHB are metabolites that are accumulated as intracellular granules, usually under conditions where there is an excess of carbon source and limitation of some nutrient such as nitrogen or phosphorus. Examples of naturally producing bacteria are *Cupriavidus necator*, *Alcaligenes latus*, *Bacillus* spp., and *Burkholderia sacchari*, and it can also be produced by recombinant bacteria such as *Escherichia coli* and even *Saccharomyces cerevisiae*.^25–27^ The PHB synthesis pathway from *C. necator*, which is one of the most well-known natural producers, is illustrated in Figure 1.

**Figure 1:**
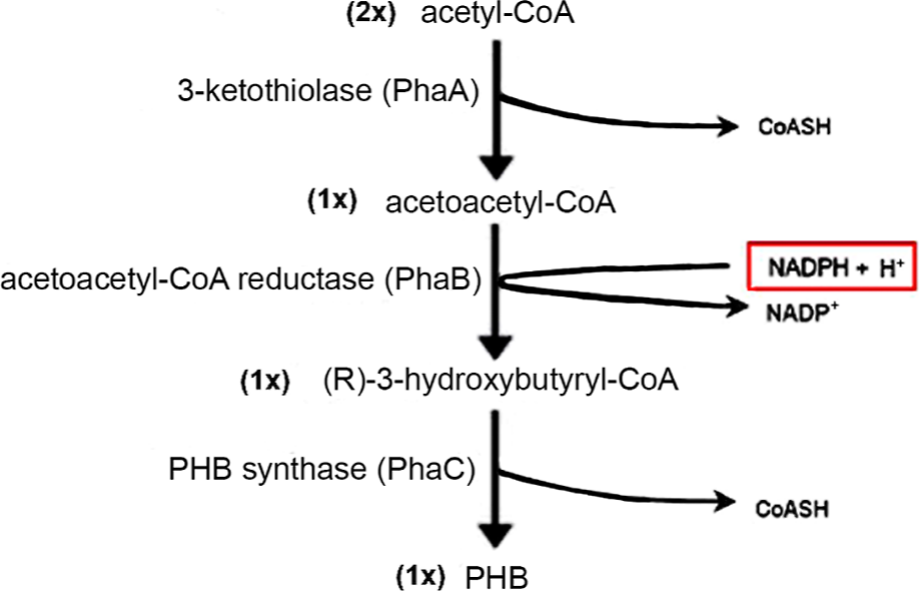
Poly-3-hydroxybutyrate synthesis pathway from *Cupriavidus necator*.

It can be seen in Figure 1 that the reaction catalyzed by the enzyme acetoacetyl-CoA reductase (PhaB) on the PHB synthesis pathway from *C. necator* is NADPH-dependent.^28–31^ However, there are reports in the literature that microorganisms such as Halomonas bluephagenesis and Candidatus Accumulibacter have a synthesis pathway with more affinity toward the NADH cofactor.^32,33^

PHB production in bacteria can be separated into two categories: those that need some nutrient limitation and the production is not associated with the growth phase, and those that do not need any nutrient limitation and the production can happen during growth.^34,35^ Natural PHB producers like *C. necator* tend to produce PHB in two stages, where the first stage is dedicated to growth, until nutrient limitation stops growth and carbon flux is then directed to PHB synthesis.^36^ Meanwhile, for recombinant *E. coli*, most reports indicate that production happens during the growth phase^37,38^ and without nutrient limitation. Nevertheless, there are some reports that highlight *E. coli* capability for both growth- and nongrowth-associated PHB production.^38^

The PHB polymer has high brittleness and melting temperature, is not soluble in water, and has material properties similar to those of polypropylene.^23,39,40^ Characteristics such as crystallinity and average molecular weight have an influence on its physical properties.^23,39,41^ It is a semicrystalline polymer because it is part crystalline and part amorphous, with its degree of crystallinity varying from 50 to 90%.^23,41^ The average molecular weight of the PHB polymer such as the ones produced by *C. necator* is in the range of 1.0 × 10^–4^ to 4.0 × 10^–6^ Da.^42–44^ Its monomer and chemical structure are listed in Figure 2.

**Figure 2:**
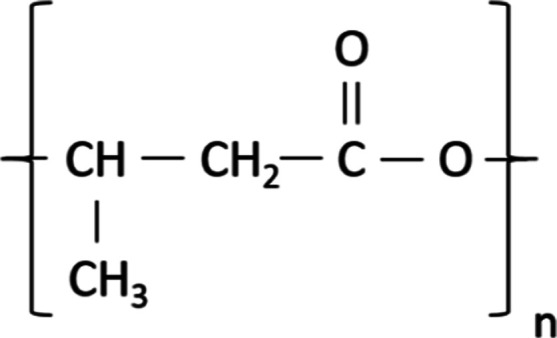
Structure of poly-3-hydroxybutyrate. Source: dos Santos et al.^23^

PHB production has been studied with classic FBA in the past and a few works where improvements in PHB production were accomplished can be cited, such as the works by Lin et al.^8^ and Zheng et al.^27^ There are also works that attempted to create mechanistic models that describe PHB production. The work by Penloglou et al.,^45^ for instance, developed a kinetic model that predicts with a satisfactory degree the intracellular accumulation profile and the molecular weight distribution of PHB. Due to the complexity of the interaction between the two types of models, there are just a few contributions that combine mechanistic/kinetic and stoichiometry for PHB production, but there are none capable of predicting the full range of properties (molar mass, dispersity, crystallinity, purity).^45–49^ Meanwhile, stoichiometric modeling such as the constraint-based FBA and its derivatives are methods that take some simplification hypotheses and account only for reaction stoichiometry and thermodynamic limitations to some degree but, thanks to that, are able to operate at full genome-scale and obtain useful information, and as such, are widely used in systems biology.^50–52^

Now, since both PHB and biomass formation share common precursors in the form of acetyl-CoA and NADPH, and given that PHB is an internal metabolite and hence the amount of PHB accumulated is linked with the amount of biomass formed, a trade-off between PHB and biomass formation can occur.^20^ With the DySEEP approach proposed in this work, the maximum PHB production potential of the three microorganisms was investigated. There are other works in the literature that present approaches to evaluate the production of growth-associated bioproducts and the trade-offs through DFBA simulations.^19,20^ But a problem with these approaches is that they fix the substrate uptake rate at the maximum value, which skews the results for growth and productivity obtained in the simulations, as they neglect potential inhibitory effects such as that of the high substrate concentration on the uptake rate and reduction of metabolic activity in the cell as a result of low substrate availability. To avoid this problem, the DySEEP approach runs the simulations directly on DFBA, where rather than having to fix the substrate uptake rate, the kinetic-like expression set in the DFBA updates the uptake rate as a function of substrate concentration and substrate inhibitory effects. Furthermore, for the metric of performance evaluation, they used simplified expressions based on weights for the yield, titer, and productivity and that do not take downstream costs and how they relate to these bioprocess parameters into consideration.^19^ Moreover, nongrowth-associated production was not investigated.^19^ The DySEEP approach presented in this work addresses both of those gaps by using an economic metric that takes into consideration the production costs, including the downstream steps, as a function of final biomass, yield, titer, and productivity of each simulation, and also presenting a method to explore the maximum potential of nongrowth-associated production, which is valuable since two-stage production processes can often present higher performance than one-stage production.^53^

The DySEEP approach allowed the identification of the scenarios that mark the start of positive cash flows, and the theoretical maximum gross profit for recombinant *C. necator* H16 with its natural NADPH-dependent PHB pathway, and for recombinant *E. coli* K-12 MG1655 and *S. cerevisiae* S288C strains harboring a NADPH-dependent PHB synthesis pathway or a NADH-dependent pathway,^32,33^ proving that the DySEEP approach can give valuable insight into potential bioprocesses of industrial interest.

## Materials and Methods

### DFBA Program, Models, and Parameters Description

In this work, an easy-to-use MATLAB program was written for the DFBA simulations that are part of the DySEEP approach. The program uses the direct approach (DA) method, which uses implicit ODE integrators with adaptive step size such as MATLAB’s ODE15s. That is because DFBA problems are often stiff, meaning fast variation of the concentrations can occur, which then requires small time steps for stability.^54,55^ There are a few available software for DFBA simulations such as DyMMM,^19^ which also used the direct approach, and DFBAlab,^54^ which uses lexicographic optimization. But to allow a more straightforward customization of the parameters in the kinetic expression and of the metabolites whose concentration would be tracked in the PHB production simulations, this new MATLAB program was used instead.

To show how the proposed approach can be applied, the production of PHB was used as a case study. In order to perform a simulation, a metabolic model, the initial substrate and biomass concentrations, and the external metabolites to be tracked must be set. Initial glucose and biomass concentrations were 25 g/L and 0.25 g/L, respectively (in a working volume of 200 m^3^). To have the simulations on the same basis, allowing proper comparisons, all simulations use the same adopted total amount of glucose of 5000 kg. The metabolites tracked were glucose, ammonia, PHB, acetate, lactate, ethanol, formate, and succinate. The MATLAB program uses linprog to solve the linear optimization problem and ODE15s to solve the system of differential mass balance equations. The full program, together with instructions on how to use it and the metabolic models used, is available in the Supporting Information A.

The metabolic models used in the simulations were *E. coli* iML1515,^56^ *C. necator* RehMBEL1391,^57,58^ and *S. cerevisiae* iMM904.^59^ The scripts for adding the relevant metabolites and for adding the PHB synthesis pathway in the *E. coli* and *S. cerevisiae* models, since they are not naturally PHB producing microorganisms, and the glucose transport system in the *C. necator* model, since *C. necator* H16 cannot naturally grow on glucose but can be engineered to be capable of accomplishing that,^60^ are available in the Supporting Information B. The PHB synthesis pathway added considers the production of the PHB monomer, with chemical formula C_4_H_6_O_2_ and molecular mass 86 g/mol.^40,61^ For *E. coli*, the growth- and nongrowth-associated production under aerobic and anaerobic conditions with a NADPH- and a NADH-dependent pathway was explored. For *C. necator*, the nongrowth-associated PHB production with aerobic growth and production phase and with aerobic growth and anaerobic production phase was explored. Anaerobic growth phase was not explored because *C. necator* cannot grow on full anaerobiosis according to model RehMBEL1391. Glucose was used rather than fructose, which is *C. necator* H16 preferred substrate^60,62^ so that all microorganisms tested in the simulations are on the same basis, allowing for comparisons. For *S. cerevisiae*, the nongrowth-associated aerobic conditions with a NADPH- and a NADH-dependent pathway were explored. Anaerobic conditions were not explored because modifications to the model iMM904 are required for anaerobic simulations.

There is a limited space inside a cell for the accumulation of PHB, and because of that, the concept of PHB content becomes important.^63–65^ Total biomass is defined as the sum of residual biomass and PHB mass, and PHB content is the ratio between PHB mass and total biomass, usually expressed as wt %.^66^ The literature points a realistic maximum PHB content of around 85%.^8,35,67^ However, FBA simulations are based on the premise of steady state for intracellular fluxes, meaning there is no accumulation of intracellular metabolites in an FBA simulation.^22^ To simulate PHB production in FBA/DFBA then, it is necessary to add an artificial PHB exporting reaction, where PHB is transported from inside the cell to the exterior. This is an artificial reaction because it does not exist in vivo, similar to how biomass formation is represented through an artificial biomass reaction in genome-scale models.^68^ But despite that, it is still possible to estimate how much PHB content the PHB excreted would represent, in case it was inside the cell as it is in vivo. By adding the cell mass and the PHB mass produced in a simulation, an equivalent to the previously defined total biomass is obtained.

DFBA uses kinetic expressions to describe the substrate uptake rate. The parameters of these kinetic equations need to be adjusted with data from the literature depending on which organism is being used in the simulation. The kinetic expression used to describe the glucose uptake was a modified Michaelis–Menten kinetics equation that takes into consideration substrate inhibition, as shown in eq 1.^69^

1 $$\nu_{glc}\left(t\right)=-\nu_{glc,\max}\frac{C_{glc}\left(t\right)}{C_{glc}\left(t\right)+K_{glc}+\frac{C_{glc}{\left(t\right)}^{2}}{K_{i,glc}}}$$

where ν_glc_(*t*) is the glucose uptake rate at any given time, ν_glc,max_ is the maximum glucose uptake found in the literature, *C*_glc_(*t*) (mmol/L) is the glucose concentration at any given time, *K*_glc_ (mmol/L) is the saturation constant for glucose transport, and *K*_*i*,glc_ (mmol/L) is the glucose inhibition constant. When not directly available in the literature, the glucose inhibition constants were estimated based on experimental data found in the literature, such as glucose concentration above 16 g/L starting to inhibit *E. coli* growth^70^ and above 20 g/L starting to inhibit *C. necator* growth.^71^

No oxygen uptake kinetics was considered since it is assumed for all the simulations that the oxygen concentration in the reactor is controlled and kept constant. So the oxygen uptake considered is the maximum oxygen uptake rate of each of these microorganisms. Table 1 presents the parameters for eq 1 (maximum glucose uptake rates and saturation and inhibition constants) and the maximum oxygen uptake rates used in the simulations.

**Table 1:** Parameters Used for the DFBA Simulations

name	aerobic ν_glc,max_	anaerobic ν_glc,max_	$\nu_{O_{2},\max}$	*K*_glc_	*K*_*i*,glc_	source
	(mmol/gCDW h)	(mmol/gCDW h)	(mmol/gCDW h)	(mmol/L)	(mmol/L)	
*E. coli*	10.5	18.5	15	0.015	8,941.7	^22^
*C. necator*	3		5	0.015	11,139.0	^58^
*S. cerevisiae*	22.5		1.5	4.884	27,102.6	^72^

The internal flux distribution for each time step is found by maximizing the objective function of the linear optimization problem derived from FBA, which can be mathematically described, as shown in eq 2.

2 max⁡⁣fTνs.t.⁣Sν=0νmin≤ν≤νmax

where *f* is the objective function, ν is the flux vector, and *S* is the *mxn* stoichiometric matrix, where *m* is the number of metabolites and *n* the number of reactions, and ν_min_ and ν_max_ are the vectors containing the minimum and maximum flux each reaction can assume, respectively.

The concentration of the external metabolites over time in a batch system is described according to eqs 3–5.

3 $$\frac{dX}{dt}=\mu \;X$$

4 $$\frac{dS}{dt}=-\nu_{S}\;X$$

5 $$\frac{dP}{dt}=\nu_{P}\;X$$

where μ is the specific growth rate (h^–1^), *X* is the biomass concentration (gCDW/L), *S* is the substrate concentration (mmol substrate/L), ν_*S*_ is the substrate uptake rate (mmol substrate/gCDW h), *P* is the product concentration (mmol product/L), and ν_P_ is the product production rate (mmol product/gCDW h).

### Growth-Associated PHB Production Simulations

Using the MATLAB program, a series of simulations exploring the trade-off between biomass and PHB production were carried out to analyze the potential for growth-associated with PHB production with *E. coli*. The simulations have growth as the objective function, but with each simulation, a greater flux for the PHB production reaction is fixed, going from zero up to the maximum possible flux to PHB given the glucose uptake kinetics and the reactions in the metabolic model. For each simulation, the resulting biomass concentration, PHB yield, PHB titer, and operation time *t*_(op)_ obtained were registered and evaluated. The procedure used for the growth-associated PHB production simulations is summarized in Figure 3, the script for calculation of the bioreactor operation costs is available on Supporting Information D, and the spreadsheets for total production costs calculation are available on Supporting Information F.

**Figure 3:**
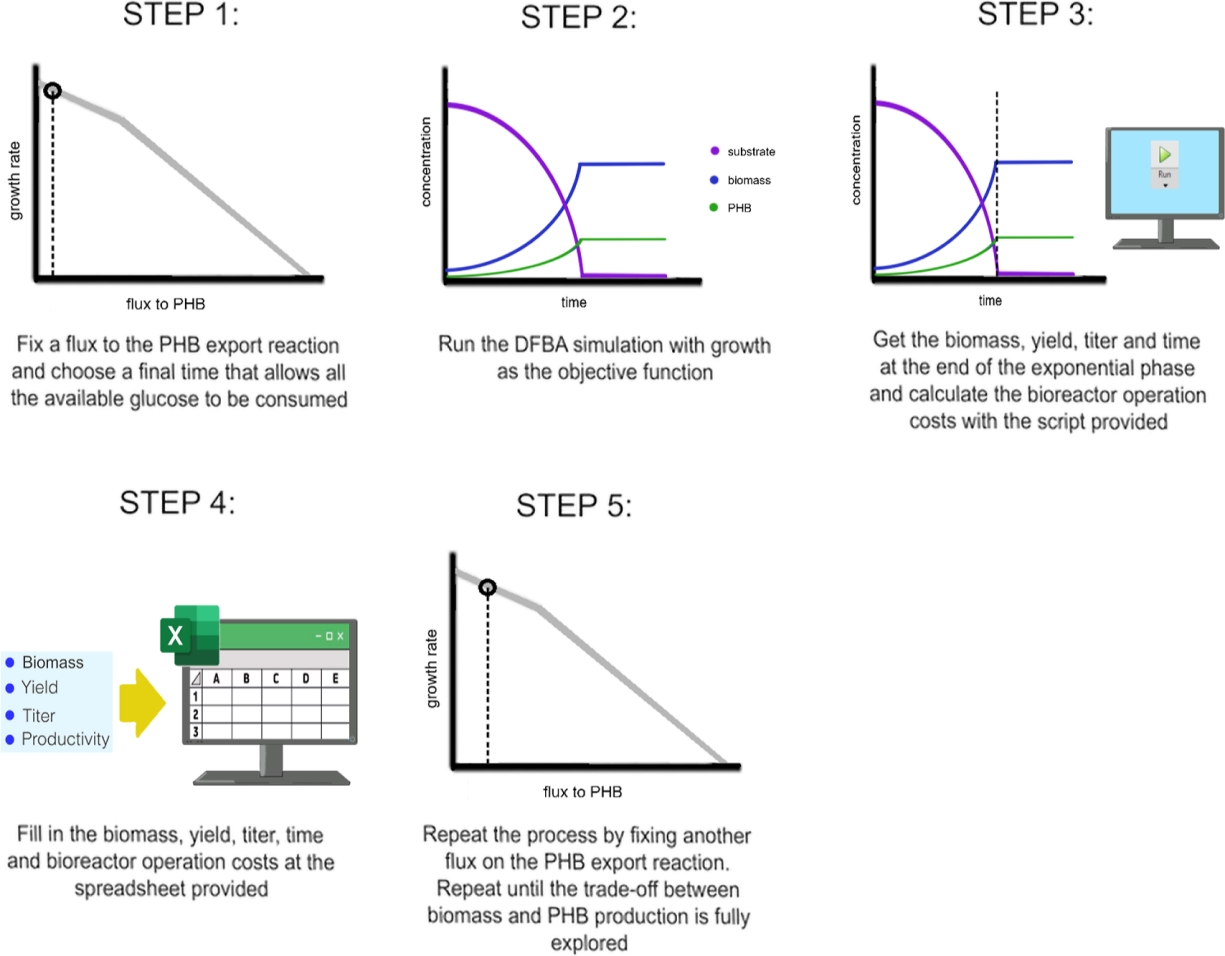
Procedure used for the growth-associated simulations.

### Nongrowth-Associated PHB Production Simulations

The nongrowth-associated PHB production simulations, carried out for *E. coli*, *C. necator*, and *S. cerevisiae*, were split into two parts, one where the objective function is growth, and then followed by one where the objective function is PHB production. This represents a process where first there is a growth-dedicated phase, followed by a dedicated PHB production phase triggered by limitation of nutrients such as nitrogen or by the use of genetic toggle-switches.^7,73,74^ In this scenario, exploration of how much of a given total amount of glucose should be allocated to each phase in order to maximize the performance can be done with the DySEEP approach. A series of simulations were carried out varying the percentage of the total glucose that is allocated to the growth phase, with the remaining being allocated to the production phase, going from one extreme of almost 100% of the total glucose to the growth phase and close to zero to the PHB phase, all the way to the other extreme, where 0% of the total glucose is used in the growth phase and 100% is used in the PHB phase. By variation of how much of a given amount of glucose is allocated to each phase, the ideal switch point can be found. The procedure used for the nongrowth-associated PHB production simulations is summarized in Figure 4, the script for calculation of the bioreactor operation costs is available in the Supporting Information D, and the spreadsheets for total production costs calculation are available on the Supporting Information I.

**Figure 4:**
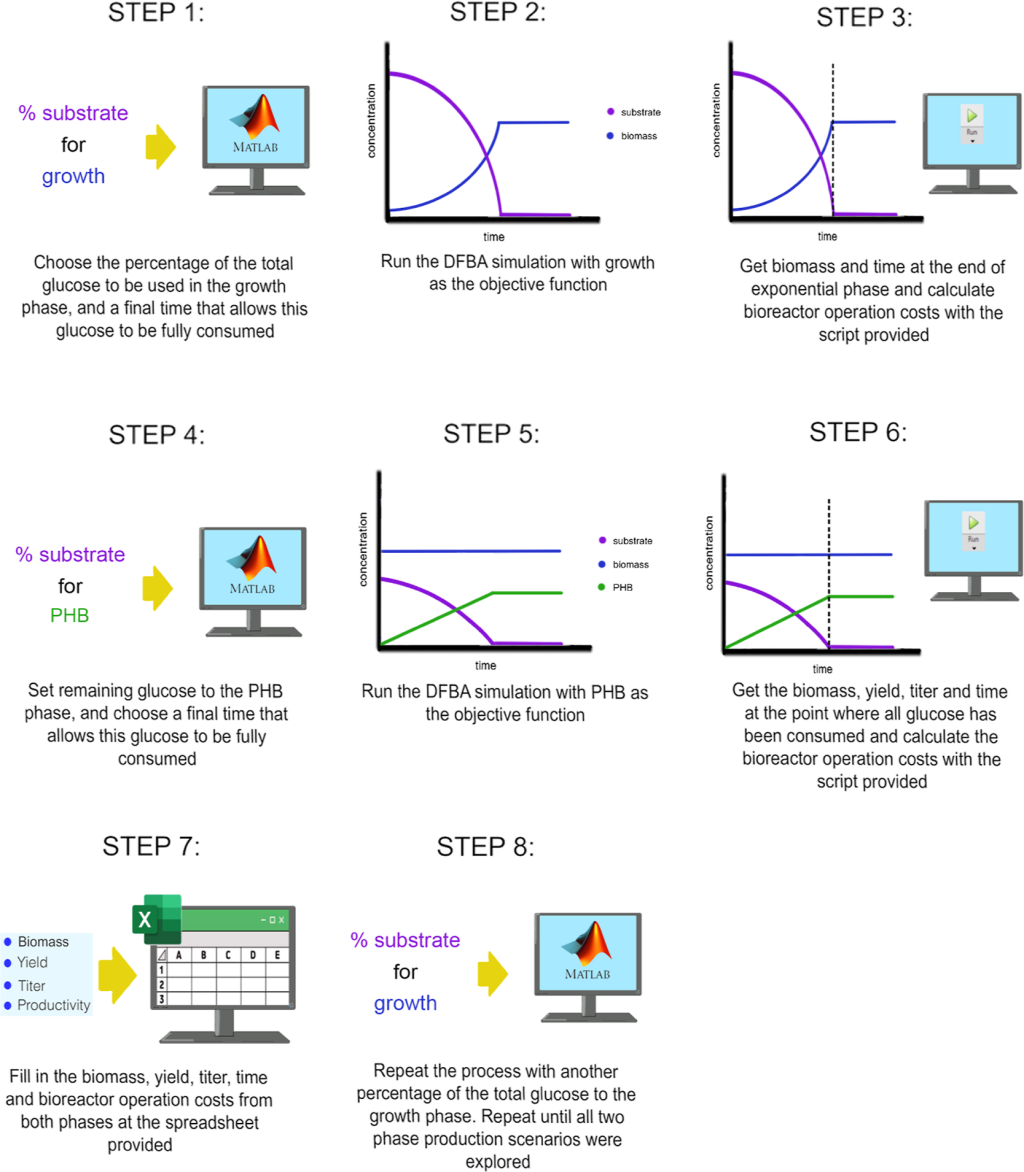
Procedure used for the nongrowth-associated simulations.

### Determining the Monthly Gross Profit

The proposed metric to evaluate the performance, in the case of a pre-existing plant, is the monthly gross profit. This work presents a relatively straightforward method to estimate the monthly gross profit using design relations found in the literature together with the parameters obtained in the simulations. Essentially, the monthly gross profit can be expressed as a function of the final biomass, product yield, titer, and productivity obtained from each simulation. With eq 6, the gross profit of a process can be calculated.^75^

6 GP=R−(RM+OP+US+DS)

where GP is the gross profit, *R* is the revenue obtained from the product sales, RM is the raw material cost, OP is the reactor operational cost, US is the upstream expenses, and DS is the downstream expenses. The selling price of PHB used in the simulation was USD 5.5/kg.^76^ Usually, not all polymer produced is recovered, and often there are some impurities together with the polymer. Therefore, the usual PHB recovery of 97% and PHB purity of 98% were taken into consideration for the calculation of the revenue with PHB sales.^7^ But it is worth highlighting that other factors can affect the properties and the economics of PHB production such as the degree of crystallinity and the molecular weight of the polymer produced, which cannot be predicted with stoichiometric modeling such as used by FBA/DFBA simulations. Therefore, given that this work uses stoichiometric modeling and given that no current mechanistic model available can predict all PHB properties,^45–49^ as discussed in the Introduction section, this work must assume as a simplification hypothesis that 98% of the PHB produced (the percentage of the PHB produced that is pure PHB) matches the desired properties well enough and can be sold by the chosen market price. Table 2 shows the costs for the feedstock and utilities used for the simulations.

**Table 2:** Cost of Feedstock and Utilities Used for the Simulations

item	cost	source
glucose	USD 350/ton	^77^
steam	USD 4/ton	^76^
electricity	USD 126/MWh	^78^
solvent	USD 1.15/L	^76^

The upstream cost was simplified as the cost of medium and sterilization, and since the type and volume of medium used in all simulations are the same and not a function of biomass, PHB yield, titer, and productivity, the upstream cost was treated as a constant. The work by Cardoso et al.^78^ provides a table with the prices for many compounds used in bacterial culture media. Assuming a mineral medium with the composition as presented in Table 3, the cost of the 200 m^3^ medium used can be calculated.

**Table 3:** Mineral Medium Composition^a^

compound	concentration (g/L)
Na_2_HPO_4_	3.5
KH_2_PO_4_	1.5
${\left({NH}_{4}\right)}_{2}{\mathrm{SO}}_{4}$	1.0
citr. Fe·NH_4_	0.06
MgSO_4_·7H_2_O	0.20
CaCl_2_·2H_2_O	0.01
kanamycin	0.05

a Source: Ramsay et al.^79^

Calculation of sterilization cost was done by estimating how much steam would be needed to heat the medium from 25 to 121 °C, usual temperature used for sterilization.^80^

In simple terms, the reactor operation costs can be broken down into aeration costs (in the case of the aerobic process), agitation costs, and cooling costs. The work by Cardoso et al.^78^ presents a methodology to estimate the operational costs of bioprocesses with a few design equations. These equations were adapted to the data available and information that can be retrieved when running DFBA simulations. The aeration costs are mainly related to the compressor power consumption. The oxygen demand changes throughout the culture as the biomass increases; therefore, in order to keep a constant oxygen concentration in the medium, the aeration has to increase accordingly. Also, aeration systems usually have low oxygen transfer efficiency from the bubble to the medium, around 20%,^68,81^ which was used together with the biomass concentration over time, the maximum oxygen uptake rate of the cell, and the air composition to calculate the necessary air flow rate in the inlet over time for the aeration (Supporting Information C). The compressor power consumption is given in eq 7.

7 $$P_{C}\left(t\right)=\frac{P_{in}Q_{air,in}\left(t\right)}{n_{c}}\frac{\gamma }{\gamma -1}{\left(\frac{P_{reac}}{P_{in}}\right)}^{\left(\left(\frac{\gamma -1}{\gamma }\right)-1\right)}\frac{1}{1000}$$

where *P*_C_(*t*) is the compressor power consumption (kW) at any given time, *P*_in_ is the inlet pressure, which is the ambient pressure of 101,325 Pa, *Q*_air,in_(*t*) is the air flow rate in the inlet at any given time (m^3^/s), *P*_reac_ is the reactor pressure of 263,200 Pa, γ is the isentropic exponent (1.4), and *n*_c_ is the efficiency number of the compressor (0.7).

Agitation in a bioreactor is used with the goal of keeping the homogeneity of the medium and to help with the oxygen transfer rate. The agitation has to increase together with the increasing oxygen demand due to biomass formation throughout the culture in order to keep the oxygen concentration in the medium constant. With eq 8, the power required for stirring can be estimated.^82^

8 $$k_{L}a\left(t\right)=0.002\;{\left(\frac{P_{S}\left(t\right)}{V}\right)}^{0.7}{V_{super\left(t\right)}}^{0.2}$$

where *k*_L_ *a* is in s^–1^, *P*_S_(*t*) is the gassed stirring power consumption (W) at any given time, and *V* is the bioreactor volume (m^3^). Rearranging eq 8, *P*_S_(*t*) can be calculated.

In the case of anaerobic culture, agitation is used mostly just to keep homogeneity; therefore, there is no need for a control system as a function of the *k*_L_ *a*(*t*), and the agitation can be less intense than the aerobic cultures with the intention of driving down operational costs. Therefore, adopting an agitation of 50 rpm (0.83 s^–1^),^83–86^ the ungassed stirring power required can be estimated with eq 9.^87–91^

9 $$P_{S}=c\;\rho_{medium}N^{3}d^{5}$$

where *c* is the power number (*a* value of 4, for the conditions simulated), ρ_medium_ is the medium density (1032 kg/m^3^), *N* is the chosen agitation (0.83 s^–1^), and *d* is the bioreactor diameter (5 m).

Temperature control in a bioprocess is necessary because cell metabolism releases heat. The heat released by the cell can be related to the oxygen uptake rate as a function of time (OUR­(*t*) in mmol/L h), according to eq 10, in the case of an aerobic culture.

10 $$Q_{heat}\left(t\right)=K\;OUR\left(t\right)\;V$$

where *Q*_heat_(*t*) is the metabolic heat release rate at any given time (kJ/h) and *K* is a constant of proportionality of 0.50 kJ/mmol O_2_.

In the case of an anaerobic culture, rather than estimating the metabolic heat released as a function of the oxygen consumed, the heat generated can be related to the amount of glucose consumed. For this work, a relation of 235 kJ per mol of glucose consumed was used.^86^ Therefore, the heat released due to metabolic activity in an anaerobic culture can be estimated by using eq 11.

11 $$E_{M}=\frac{K_{anae}\;S_{\left(cons\right)}V}{3600}$$

where *K*_anae_ is the constant of proportionality of 235 kJ/mol glc and *S*_(cons)_ is the total substrate consumed divided by the reactor volume (mol glc/L).

The detailed description of how the aeration, agitation, and cooling costs were calculated is available in Supporting Information C. Now for downstream, the following main steps were considered: homogenization, first centrifugation, extraction, washing and centrifugation, washing and centrifugation once again, drying, and water and solid waste treatment.^92^ Table 4 shows the relations used to estimate the costs or utilities demand for each of these steps as a function of the biomass concentration, culture volume, and PHB yield, titer, and productivity obtained with each simulation. In addition, while there are other possible downstream configurations for PHB production in the industry, for the purposes of this work, which is to illustrate how the DySEEP approach can be used to explore the production potential of bioprocesses and identify potential targets for metabolic engineering and synthetic biology strategies, testing only the chosen downstream configuration is enough.

**Table 4:** Cost and Utilities Demand for the Main PHB Production Process Downstream Steps

downstream step	cost	source
homogenization	0.35 kWh/kg CDW	^92^
centrifugation	0.5 kWh/m^3^	^93^
extraction	9.25 L solvent/kg CDW	^76^
drying	2 kg steam/kg PHB	^94^
wastewater treatment	USD 0.5/m^3^	^77^

The detailed description of all the steps used to estimate the costs of upstream, bioreactor operation (aeration, agitation, and cooling), and downstream is available in the Supporting Information C, and MATLAB scripts to calculate the bioreactor operation costs using the described equations and the parameters obtained with each simulation are available in the Supporting Information D. After estimating the costs with upstream, bioreactor operation, and downstream, the next step is to estimate how many batches per month are possible, for each simulation. The operation time *t*_(op)_ of a batch is the time required to reach the final PHB titer obtained in that batch. The turnaround time *t*_(off)_, which is the time needed for cleaning, preparation, and starting another batch, was assumed to be 12 h.^95^ The average month duration time was adopted as 30 days, equivalent to 720 h. Thus, the number of batches that can be carried out in one month (*N*_bat_) can be calculated according to eq 12.

12 $$N_{bat}=\frac{720}{t_{\left(op\right)}+t_{\left(off\right)}}$$

With the gross profit per batch and the number of batches that can be done per month, the monthly gross profit can be calculated using eq 13.

13 $$MGP=GP\;N_{bat}$$

Equation 13 allows estimation in a simplified way of the gross profit obtained per month, as a function of parameters such as the biomass, product yield, titer, and operation time (and, with that, the productivity).

### Economic Analysis

To carry on the economic analysis, the main equipment for the PHB production process were considered. Prices for each of those equipment can be found in the literature,^76,77,96^ and with eqs 14 and 15, their capacity was adjusted for a bioreactor with 295 m^3^ and their prices were updated for the base year of 2022, respectively.^77^

14 $$\mathrm{Cos}t_{\left(adj,size\right)}=\mathrm{Cos}t_{\left(ref,size\right)}{\frac{Size_{\left(adj\right)}}{Size_{\left(ref\right)}}}^{0.6}$$

where Size_(ref)_ is the reference equipment size, Cost_(ref, size)_ is the cost of the reference equipment, Size_(adj)_ is the adjusted size of equipment, and Cost_(adj, size)_ is the cost of the equipment adjusted in relation to size.

15 $$\mathrm{Cos}t_{\left(final\right)}=\mathrm{Cos}t_{\left(size,adj\right)}\frac{I_{\left(2\right)}}{I_{\left(1\right)}}$$

where *I*_(1)_ and *I*_(2)_ are the Chemical Engineering Plant Cost Index (CEPCI), for the equipment in its original year and at 2022, respectively, Cost_(adj, size)_ is the cost of the equipment adjusted in relation to size, and Cost_(final)_ is the cost of the equipment adjusted in relation to both size and inflation. The CEPCI indexes are available in the *Chemical Engineering* Magazine. The CEPCI used for 2022 was that of August, which is 824.5.

With the total equipment cost, a set of average multipliers can be applied over the total equipment cost to determine the direct fixed capital (DFC) for a bioprocess.^77^ Table 5 shows the average multipliers used in this work to estimate the cost of the items that are part of the DFC and to estimate the total investment. Supporting Information presents the equipment costs and the total investment required.

**Table 5:** Average Multipliers Used to Estimate the Total Investment

item	average multiplier/calculation method
equipment purchase cost (PC)	-
	
total plant direct cost (TPDC)	-
1. installation	0.50 × PC
2. process piping	0.40 × PC
3. instrumentation	0.35 × PC
4. insulation	0.03 × PC
5. electrical	0.15 × PC
6. buildings	0.45 × PC
7. yard improvement	0.15 × PC
8. auxiliary facilities	0.50 × PC

total plant indirect cost (TPIC)	-
9. engineering	0.25 × TPDC
10. construction	0.354 × TPDC
	
total plant cost (TPC)	TPDC + TPIC
	
11. contractor’s fee	0.05 × TPC
12. contingency	0.10 × TPC
	
direct fixed capital (DFC)	TPC + 11 + 12
	
13. working capital	0.1 × DFC
14. start/validation costs	0.05 × DFC
	
total investment	DFC + 13 + 14

The economic analysis was carried out for a 10 year period of operation, adopting a tax value of 34% and a discount rate of 12%, and the depreciation was calculated using the straight-line method, assuming a salvage price of 5% of the DFC.^76,77,95^ With that, the Return On Investment (ROI), payback time, Net Present Value (NPV), and Internal Rate of Return (IRR) could be calculated. The ROI was calculated according to eq 16.

16 $$ROI=\frac{Annual\;net\;profit}{Total\;investment}100$$

where ROI is the Return on Investment (%). The payback time was estimated with eq 17.

17 $$Payback\;time=\frac{Total\;investment}{Annual\;net\;profit}$$

where payback time is in years. Equation 18 presents the Net Present Value.

18 $$NPV=\sum_{n=0}^{N}\frac{{NCF}_{n}}{{\left(1+discount\;rate\right)}^{n}}$$

where NPV is the Net Present Value in US dollars, *n* is the period in which the cash flow occurs, *N* is the holding period of investment (10 years), and NCF_*n*_ is the net cash flow in the period. Eq 19 shows how to calculate the Internal Rate of Return.

19 $$0=\sum_{n=0}^{N}\frac{{NCF}_{n}}{{\left(1+IRR\right)}^{n}}$$

where IRR is the Internal Rate of Return (%); finding it is an iterative process.

### Flux Visualization and Flux Variability Analysis

The visualization of the computed set of internal fluxes of the simulated cells can be done with web applications such as Escher-FBA^97^ and IMFLer.^98^ Both provide intuitive tools to run FBA simulations and visualize the results within a chosen manually drawn metabolic map, for instance, a map of the central carbon metabolism. The tools allow map editing, a feature that was used to add the PHB synthesis pathway to the *E. coli* metabolic map for visualization of the PHB producing *E. coli* strains simulated. FBA simulations do not often have a unique solution; that is, there may be an infinite set of internal fluxes that give the same value for the objective function, so to account for that, Flux Variability Analysis (FVA) was applied. FVA is a mathematical approach that identifies the minimum and maximum flux that each reaction can have while still leading to the same value of objective function. The IMFLer tool can also run FVA simulations or import FVA results. Therefore, the FVA simulations were carried out using COBRA toolbox for MATLAB^99^ and later imported to IMFLer. With COBRA toolbox, IMFLer, and a map of the central metabolism of *E. coli*, the flux distribution ranges for the scenario that leads to the highest monthly gross profit and the flux distribution ranges for a simulation constrained with experimental data of PHB production with recombinant *E. coli* found in the literature^25,27^ were manually compared. This comparison with the intention of finding differences in the fluxes, together with biological knowledge about the microorganism, allows the identification of key reactions to be further investigated and the suggestion of possible genetic modifications that could potentially improve growth-associated with PHB production in *E. coli*.

## Results and Discussion

### Results for the Batch Process Growth-Associated PHB Production Simulations, Assuming a Pre-existing Plant

Approaches such as flux balance analysis are often used to determine the maximum growth of an organism under different conditions or the maximum yield for a product of interest. Even though these values may not be quite achievable in practice, they are useful to set a threshold for what can be expected of the cells tested and help with an initial evaluation of the viability of a desired bioprocess and other valuable information. For instance, an FBA simulation with *E. coli* model iML1515 using realistic uptakes of glucose and oxygen reveals a maximum growth rate of 0.710 h^–1^.

In the scenario of synthesis of products that share common precursors with biomass formation and products that accumulate internally in the cell, a trade-off between product yield and biomass formation occurs, especially if the formation of such product happens during the growth phase.^20^ Poly-3-hydroxybutyrate and biomass formation share acetyl-CoA as a precursor, and in the case of *E. coli*, the literature reports that PHB production can take place even during growth and without the requirement of nutrient limitation,^35,37^ and therefore, a trade-off may occur, which in turn can affect important bioprocess parameters such as the product titer and productivity.

Furthermore, the availability of NADPH in *E. coli* may be a limiting factor for PHB production through the synthesis pathway from *C. necator*.^25,27,100^ However, the PHB synthesis pathway of bacteria such as H. bluephagenesis seems to have more affinity for the NADH cofactor than NADPH,^32^ and a recombinant *E. coli* harboring the PHB synthesis pathway from the bacteria Candidatus Accumulibacter, which was also found to have more affinity for the NADH cofactor, was successfully capable of accumulating PHB.^33^ In order to analyze the trade-off between growth and PHB yield and the effect of using a NADPH- or NADH-dependent PHB synthesis pathway in recombinant *E. coli* on aerobic or anaerobic batch cultures, DFBA simulations were carried out, and the monthly gross profit was calculated for each scenario tested. As an example, Table 6 shows the results for the simulations of growth-associated production with a NADPH-dependent pathway under aerobic conditions.

**Table 6:** Exploration of the Trade-Off between Biomass and Product Formation for *E. coli* Harboring the NADPH-Dependent PHB Synthesis Pathway under Aerobic Conditions for the Batch Process

flux to PHB	final biomass	yield	titer	productivity	PHB content	monthly profit
mmol/g h	g/L	g PHB/g glc	g PHB/L	g PHB/L h	%WT	kUSD/month
≈0	9.675	0.01	0.25	0.05	2.52	–273
1	9.42	0.05	1.15	0.22	10.85	–262
2	9.17	0.09	2.29	0.42	20.01	–229
3	8.92	0.14	3.44	0.62	27.84	–194
4	8.66	0.18	4.59	0.81	34.62	–150
5	8.15	0.23	5.74	0.97	41.32	–110
6	7.23	0.28	6.88	1.06	48.75	–65
7	6.30	0.32	8.02	1.12	56.01	–23
8	5.28	0.37	9.17	1.12	63.48	16
9	4.24	0.41	10.31	1.08	70.89	51
10	3.19	0.46	11.46	0.98	78.23	79
11	2.15	0.50	12.60	0.82	85.42	98
12	1.11	0.55	13.73	0.58	92.54	97
12.909	0.25	0.59	14.67	0.28	98.32	64

Each row in the first column in Table 6 shows how much flux to PHB synthesis was fixed in each scenario simulated, starting from almost zero flux being directed to PHB (and almost all carbon flux is directed to biomass formation), all the way to all flux being directed to PHB formation, and hence the initial biomass concentration remaining constant as no flux is directed to biomass. The values in the other columns are the result of fixing that amount of flux to PHB synthesis defined in each row. The data in Table 6 shows that the point where the revenue and expenses become equal is achieved at a PHB yield between 0.32 and 0.37 g PHB/g glc, with the cash flow being already positive at a yield of 0.37 g PHB/g glc and its consequent titer of 9.17 g PHB/L, productivity of 1.12 g PHB/L h, and PHB content of about 63 wt %, under the conditions tested. For comparison, experimental results from the shake flask or batch process available in the literature so far for PHB production with recombinant *E. coli* report yield of 0.24 g PHB/g glucose when bioreactor operation was optimized^101^ and yields of 0.31 and 0.35 g PHB/g glucose with the genetically modified strains engineered in the works by Zheng et al.^27^ and Centeno-Leija et al.,^25^ respectively. The maximum PHB yield predicted by the simulations is 0.59 g of PHB/g of glc. However, with such a yield, there is no carbon being directed to biomass formation, and that has a negative impact on the productivity to a degree that ends up also decreasing the monthly gross profit. Taking into consideration the maximum PHB content previously mentioned, the scenario with the best performance led to a monthly gross profit of 98,000 USD, and the interesting thing to notice about this scenario is that it does not have the highest PHB yield, titer, or productivity but a set of these three parameters that, when operational and downstream costs are taken into consideration, results in the maximum theoretical monthly gross profit for the conditions simulated.

While real in vivo recombinant *E. coli* cells without genetic modifications beyond the insertion of the PHB synthesis pathway are unlikely to behave like the in silico identified best scenario for *E. coli*, the identification of this best scenario, its flux distribution, and its resulting bioprocess parameters provide a lot of insight and hint at what genetic modifications and other strategies should be attempted to try to improve the production of PHB with real cells. The full tables for all growth-associated simulations (aerobic and anaerobic conditions with a NADPH- and a NADH-dependent pathway with *E. coli*) are available in Supporting Information F, and Figure 5 summarizes the scenarios that lead to the highest monthly gross profit, for each condition tested in the growth-associated simulations.

**Figure 5:**
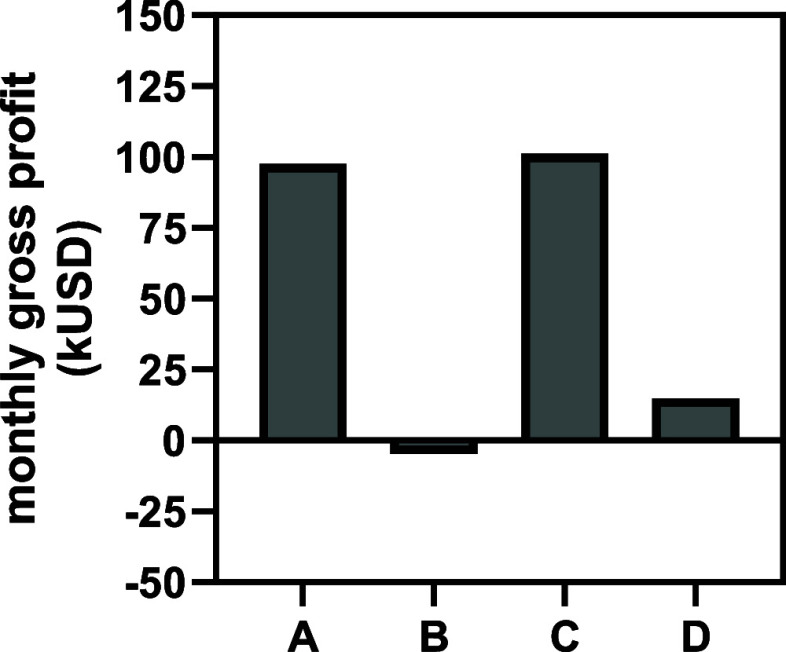
Best-performing simulations for each condition tested with growth-associated PHB production. (A) *E. coli* with the NADPH-dependent PHB pathway under aerobic conditions. (B) *E. coli* with the NADPH-dependent PHB pathway under anaerobic conditions. (C) *E. coli* with the NADH-dependent PHB pathway under aerobic conditions. (D) *E. coli* with the NADH-dependent PHB pathway under anaerobic conditions.

Comparing the results of growth-associated production under aerobic conditions for *E. coli* harboring the NADPH-dependent PHB synthesis pathway with the results for the NADH-dependent pathway, it is noted that the NADH-dependent pathway increased the maximum PHB yield from 0.59 to 0.60 g PHB/g glc (Supporting Information F). Beyond that, the Supporting Information F points out that the simulated scenario with the NADH-dependent pathway that had the best performance has the same PHB yield and titer as the one with the NADPH-dependent pathway, but greater productivity, leading to a monthly gross profit of 101,000 USD, an increase in profit of about 4% compared with the best scenario from the simulations using the NADPH-dependent PHB synthesis pathway, given the scale and conditions of these simulations. The reason for this is that, for a given flux to the PHB synthesis reaction, the FBA simulations show a higher growth rate and faster exponential phase for the model with the NADH-dependent PHB pathway than the model with the NADPH-dependent PHB pathway, which in turn has a positive effect on the productivity since productivity is related to time. A possible explanation for why *E. coli* with the NADH-dependent pathway displays this advantage can be seen in the reactions that compose the *E. coli* genome-scale model iML1515. The model shows that the *E. coli* metabolism has 24 reactions that can produce NADPH in comparison to 62 reactions that can produce NADH.

Figure 5 shows that the growth-associated anaerobic simulations with the NADPH-dependent pathway were considerably less profitable than the aerobic option, despite its lower operational costs. Comparing the maximum yield under anaerobic conditions, the NADPH-dependent pathway achieves 0.46 g of PHB/g of glc, while the NADH-dependent pathway achieves 0.51 g of PHB/g of glc (Supporting Information F). Again considering the PHB content limit, Figure 5 reveals that unlike the simulations with the NADPH-dependent pathway, a positive cash flow could be reached in the best scenario for the simulations with the NADH-dependent pathway. This shows that using a NADH-dependent pathway increased the maximum PHB production potential of *E. coli* under anaerobic conditions. Nevertheless, its performance is still below the results from the simulations under aerobic conditions; the lower operational costs were not enough to compensate for the inferior growth rate and product yield.

Estimation of the internal fluxes of cells under real experimental scenarios can be done with methods such as metabolic flux analysis (MFA) and carbon-labeled metabolic flux analysis (13C-MFA), using experimental data for external metabolites and fluxes.^102–105^ Data regarding PHB production using recombinant *E. coli* under aerobic and anaerobic batch cultures can be found in the literature.^25,27,106,107^ The literature indicates an average PHB yield of around 0.15 g PHB/g glc during aerobic batch culture^25,27,106,107^ and a PHB yield of 0.046 g PHB/g glc under anaerobic batch culture,^108^ when no further genetic modification or process optimization is carried out. For *E. coli* k-12 MG1655 specifically, the literature points a PHB yield of 0.13 g PHB/g glc and an acetate yield of 0.16 g PHB/g glc, for aerobic batch culture.^25^ Figure 6 generated using the IMFLer web application depicts the flux ranges for a simulation constrained with the mentioned experimental values for the PHB and acetate yield with *E. coli* k-12 MG1655. Versions with a zoom-in/out feature for Figure 6 and for the scenario with the NADPH-dependent pathway that results in the best performance under aerobic conditions can be seen in Supporting Information G.

**Figure 6:**
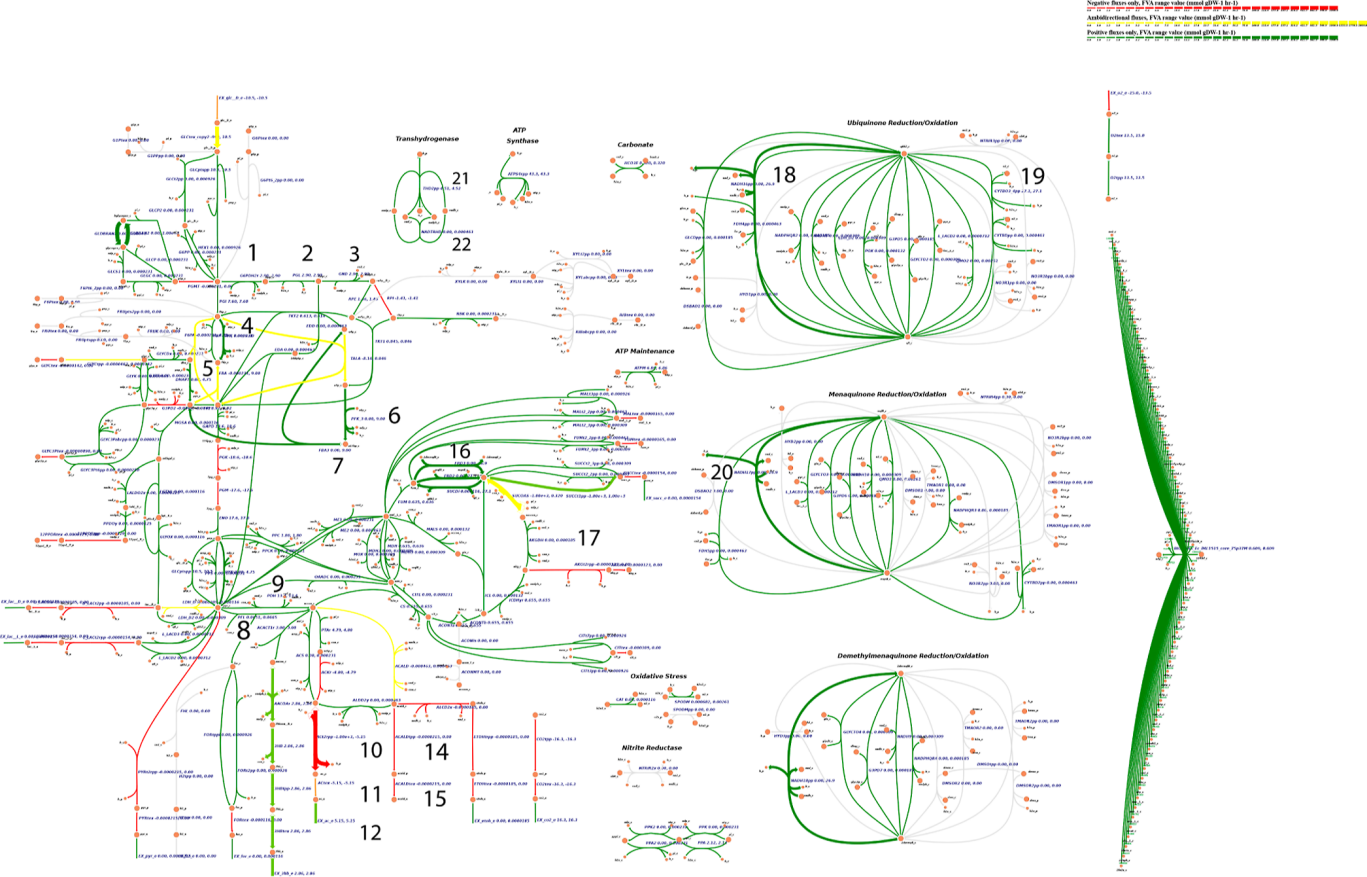
Flux ranges for *E. coli* under aerobic conditions for simulation constrained with experimental data. Green color represents positive flux, red is negative flux (reverse direction), and yellow is for ambidirectional fluxes. The numbered reactions were further investigated in Table 7. Version with zoom feature is available in Supporting Information G.

### Suggestions of Genetic Interventions for Growth-Associated PHB Production

Comparing the metabolic maps with the flux distribution ranges of the simulated scenario that leads to the best performance with the NADPH-dependent pathway under aerobic conditions and the flux distribution ranges of a simulation with *E. coli* harboring the PHB synthesis pathway from *C. necator* constrained with experimental data (Supporting Information G and Figure 6) allows the suggestion of genetic modifications that could potentially improve growth-associated PHB production in *E. coli*. The idea is to bring the metabolic flux distribution of a real cell closer to that of the scenario whose set of biomass, PHB yield, titer, and productivity leads to the highest monthly gross profit. Table 7 gives the results of the FVA simulations that represent *E. coli* without further genetic modifications under a real experimental scenario and *E. coli* harboring a NADPH-dependent PHB synthesis pathway under aerobic conditions and growth-associated production that would lead to the best economic performance. The tables with the results of the FVA simulations for the other scenarios are available in the Supporting Information H.

**Table 7:** Flux Variability Analysis Results for Experimental Data Constrained and for Best-Performing *E. coli* Simulations with NADPH-Dependent PHB Synthesis Pathway under Aerobic Conditions

map identification	reaction name	simulation constrained with experimental data		simulation with best performance	
number	in model	minimum flux	maximum flux	minimum flux	maximum flux
1	G6PDH2r	2.90	2.90	0.00	0.00
2	PGL	2.90	2.90	0.00	0.00
3	GND	2.90	2.90	0.00	0.00
4	PFK	0.00	9.00	0.00	4.31
5	FBA	0.00	9.00	0.00	4.31
6	PFK_3	0.00	9.00	0.00	4.31
7	FBA3	0.00	9.00	0.00	4.31
8	PFL	0.07	0.07	0.27	0.27
9	PDH	13.39	13.39	3.95	3.95
10	ACt2rpp	–1,000.00	–5.15	–1,000.00	0.00
11	ACtex	–5.15	–5.15	0.00	0.00
12	EX_ac_e	5.15	5.15	0.00	0.00
13	ACALD	0.00	0.00	9.08	9.34
14	ACALDtpp	0.00	0.00	0.00	0.00
15	ACALDtex	0.00	0.00	0.00	0.00
16	FRD2	0.00	27.07	0.00	13.91
17	AKGDH	0.00	0.00	0.00	0.00
18	NADH16pp	0.00	26.86	0.00	13.86
19	CYTBO3_4pp	27.06	27.06	13.91	13.91
20	NADH17pp	0.00	26.86	0.00	13.86
21	THD2pp	4.51	4.52	13.61	13.61
22	NADTRHD	0.00	0.00	0.00	0.00

The map numbers in the first column of Table 7 are just identifications to help spot these reactions in the metabolic map presented in Figure 6. The results in Table 7 indicate that some of the genetic modifications that could potentially bring the internal flux distribution closer to that of the scenario leading to the best performance for growth-associated production are as follows. Interruption of the reactions of the oxidative phase of the PP pathway (reactions with map identification numbers 1, 2, and 3), which is surprising since these are the reactions of the PP pathway that produce NADPH but probably because that comes at the cost of carbon loss due to carbon dioxide formation. Therefore, the FVA simulation predicts that no flux in the oxidative phase of the PP pathway and NADPH regeneration in other reactions of the metabolism such as with high flux in the membrane-bound transhydrogenase reaction leads to the scenario with the best performance. FVA indicates that a minimum flux of 13.61 mmol/gCDW h in the membrane-bound transhydrogenase reaction (map identification number 21) is required in the scenario that would lead to the best performance, and to achieve that in vivo, overexpression of the *pntAB* operon may be required, and there are reports in the literature where overexpression of *pntAB* indeed improved PHB production in recombinant *E. coli*.^8,27^ The FVA analysis also points out that a minimum flux of 0.27 and 3.95 mmol/gCDW h for the reactions catalyzed by the pyruvate formate lyase (PFL, map identification number 8) and pyruvate dehydrogenase enzymes (PDH, map identification number 9), respectively, is required to achieve flux distributions that lead to the best performance. It is believed, however, that for *E. coli* in vivo, the PFL is active during anaerobic conditions and inactive during aerobic conditions while PDH is active under aerobic conditions, which means that a suggestion such as forcing some flux on both may be hard to achieve in practice. Still, there are a few reports in the literature that point some level of PFL expression in aerobic conditions.^109^ Acetate excretion should be interrupted (reactions with map identification numbers 10, 11, and 12) and a minimum flux in the reversible reactions catalyzed by the phosphotransacetylase and acetate kinase enzymes, but in the direction of acetyl-CoA formation, is required. Interruption of the main acetate production pathway will have a negative effect on growth, as predicted by the simulations, but as long as the minimum growth rate predicted for the scenario that leads to the best performance (0.152 h^–1^, Supporting Information G) is achieved, the highest monthly gross profit for growth-associated production would be met. Impairment of acetate production and increase of conversion of acetate to acetyl-CoA is in line with some successful strategies to increase acetyl-CoA availability and to improve PHB production in *E. coli*.^25,110,111^ Similarly, acetaldehyde excretion should be interrupted (reactions with map numbers 14 and 15) and a minimum flux of 9.08 mmol/gCDW h in the direction of acetyl-CoA formation on the reversible reaction catalyzed by the acetaldehyde dehydrogenase (map number 13), is also required. In the TCA cycle, the reaction catalyzed by the 2-oxoglutarate dehydrogenase enzyme (map identification number 17) should be interrupted. This suggestion falls in line with what was proposed in the work by Zheng et al.,^27^ where downregulating the TCA cycle to increase acetyl-CoA availability improved PHB production in recombinant *E. coli*. Also, like in the case of interruption of acetate production, downregulation of the TCA cycle will have a negative effect on growth, but as long as the minimum growth rate predicted for this scenario is met, the highest monthly gross profit for growth-associated with production would be achieved.

### Results for the Batch Process Nongrowth-Associated PHB Production Simulations, Assuming a Pre-existing Plant

Now, while recombinant *E. coli* can produce PHB even during growth,^37^ naturally PHB producers such as *C. necator* tend to produce PHB mostly in scenarios where the growth becomes hindered by limitation of some nutrient. Furthermore, works where recombinant *S. cerevisiae* capable of producing PHB were engineered, also point to nongrowth-associated production.^26,112^ One of the strategies used in the industry for large-scale PHB production is a two-phase cultivation method, where the first stage is focused on bacterial growth by providing the ideal conditions for growth and then a second phase focused on PHB production which is usually achieved by limiting nitrogen availability. Finding an ideal switch point between the two phases can increase the profitability of the process.^7,71,111,113^ The review by Zhang et al.^114^ highlights some of the works where experiments testing different carbon to nitrogen feed ratios were carried out for PHB production, although none of them seem to use an economic metric as a function of all the main bioprocess parameters for more accurate evaluation of performance. Furthermore, there are some conflicting results in the literature, with works such as Garcia-Gonzalez et al.^115^ pointing that it is better to limit nitrogen early in the culture, while works such as Mozumder et al.^71^ indicate that improved production is obtained by limiting nitrogen later in the culture, when a high cell concentration has already been achieved. It is therefore interesting to investigate the production of PHB in scenarios where there is first a phase dedicated to growth, followed by a phase dedicated to PHB production, analyze how this affects the profitability, and also identify the ideal switch point.

To be comparable with the growth-associated with PHB production simulations, the total amount of glucose used was the same. Table 8 shows, as an example, the results for the nongrowth-associated simulations for *E. coli* cells with the NADPH-dependent pathway under aerobic conditions, and the complete tables for all nongrowth-associated simulations for *E. coli* (aerobic and anaerobic with NADPH and NADH-dependent pathway), *C. necator* (aerobic growth and production phase, and aerobic growth phase with anaerobic production phase), and *S. cerevisiae* (aerobic with NADPH and NADH-dependent pathway) are available in Supporting Information I.

**Table 8:** Exploration of Possible Two-Phase PHB Production Scenarios for *E. coli* Harboring the NADPH-Dependent PHB Synthesis Pathway under Aerobic Conditions for the Batch Process

glucose for growth phase	final biomass	yield	titer	productivity	PHB content	monthly profit
% mol of total glucose	g/L	g PHB/g glc	g PHB/L	g PHB/L h	%WT	kUSD/month
≈100	9.68	0.01	0.25	0.05	2.52	–274
90	8.73	0.06	1.47	0.28	14.39	–257
80	7.79	0.12	2.93	0.56	27.36	–211
70	6.84	0.18	4.40	0.83	39.15	–160
60	5.90	0.23	5.86	1.09	49.85	–108
50	4.96	0.29	7.33	1.31	59.68	–51
40	4.01	0.35	8.80	1.48	68.67	4
30	3.07	0.41	10.27	1.56	76.97	57
20	2.13	0.47	11.74	1.46	84.62	102
10	1.19	0.53	13.20	1.07	91.71	125
0	0.25	0.59	14.67	0.28	98.32	64

Each row in the first column in Table 8 shows how much of the total glucose was used for the growth phase and, by extension, how much glucose was used for the PHB production phase in each scenario simulated since the sum of both is equal to the total amount of glucose available. The values in the other columns are the result of using the amount of glucose for the growth phase defined in each row. The first row is a scenario where almost all glucose is used in the growth phase, and the last row is a scenario where all glucose is used in the PHB production phase and there is no growth phase, and hence, the initial biomass stays constant. Table 8 shows that for nongrowth-associated PHB production with a NADPH-dependent pathway under aerobic conditions, the point where cash flow starts to become positive is achieved when the equivalent to around 50 to 60%(mol) of the total glucose is consumed in the PHB production phase. And within the maximum PHB content, the best two-phase process in Table 8 is the one where 20% (mol) of the total glucose is used for growth and the remaining 80% (mol) for the PHB production phase, leading to a monthly gross profit of 102,000 USD. That is an increase of about 4.25% in profit compared with the best simulated cell harboring the NADPH pathway under aerobic conditions from the growth-associated production simulations (Table 6). Figure 7 summarizes the scenarios that lead to the highest monthly gross profit for each condition tested in the nongrowth-associated simulations for each microorganism, and the complete results are available in Supporting Information I.

**Figure 7:**
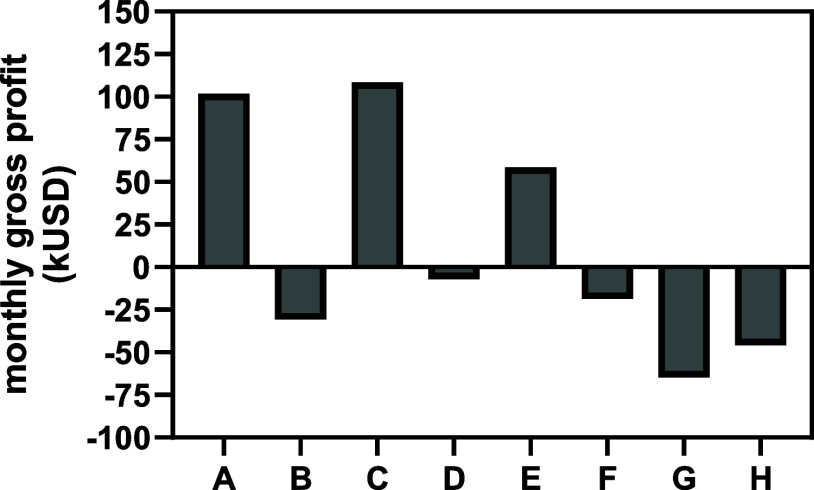
Best-performing simulations for each condition tested with nongrowth-associated PHB production. (A) *E. coli* with the NADPH-dependent PHB pathway under aerobic conditions. (B) *E. coli* with the NADPH-dependent PHB pathway under anaerobic conditions. (C) *E. coli* with the NADH-dependent PHB pathway under aerobic conditions. (D) *E. coli* with the NADH-dependent PHB pathway under anaerobic conditions. (E) *C. necator* under aerobic conditions. (F) *C. necator* with growth under aerobic conditions and the PHB production phase under anaerobic conditions. (G) *S. cerevisiae* with the NADPH-dependent PHB pathway under aerobic conditions. (H) *S. cerevisiae* with the NADH-dependent PHB pathway under aerobic conditions.

The results in Figure 7 reinforce that for *E. coli*, anaerobic PHB production is an inferior option compared with an aerobic process, even when using a NADH-dependent pathway under the conditions simulated. As for two-phase production under aerobic conditions with *E. coli* harboring a NADH-dependent pathway, the best result is also achieved when 20% (mol) of the total glucose is used for the growth phase, and 80% (mol) in the PHB production phase, leading to a monthly gross profit of 108,000 USD (Supporting Information I), which is a 7.15% increase in profit in relation to the best simulated *E. coli* harboring the NADH-dependent pathway under aerobic condition from the growth-associated PHB production simulations. Given the scale of the simulations and respecting a realistic maximum PHB content, that seems to be the scenario whose combination of final biomass, yield, titer, and productivity leads to the best results, when all production costs, revenue with PHB sales, and number of batches per month are considered.

For two-phase production, the scenarios that lead to a positive cash flow (Supporting Information I) and the scenario that leads to the highest monthly gross profit were also identified for *C. necator*. The highest monthly gross profit for *C. necator* is 59,000 USD, as indicated in Figure 7. Meanwhile, the simulations with *C. necator* where the PHB phase was carried out under anaerobic conditions could not achieve positive cash flows (Figure 7), due to low yields (Supporting Information I). Figure 7 also shows that it was not possible to achieve a positive cash flow with *S. cerevisiae* in the scale and conditions simulated. And the reason for that, given the *S. cerevisiae* model iMM904, is because the PHB yields that *S. cerevisiae* can achieve are too low and because its slow growth rate at the higher yields leads to high batch times, and by extension, low productivities (Supporting Information I).

The results in Table 8, Figure 7, and Supporting Information I seem to support the findings by Garcia-Gonzalez et al.,^115^ showing that a somewhat early shift from growth to production phase leads to better performance. But that is presuming that, after the shift, metabolic flux will indeed be primordially directed to PHB synthesis. While nutrient limitation is the most common strategy to trigger the shift to PHB synthesis so far, novel works in the field of synthetic biology where genetic toggle-switches are being developed could help ensure that the shift from growth to PHB synthesis happens when and as intended.^73,74,116–124^ Those works have explored different triggers for the genetic toggle-switch such as glucose starvation, addition of a cheap inducer in the medium, and temperature control. A few examples where genetic toggle-switches have been applied to PHB production can be given.^117,119,122^ The work by Li et al.^122^ tested different times to trigger the shift, and their results are in accordance with the idea that an early shift to PHB production led to the best results.

### Results of the Economic Analysis for a New Plant

While the estimation of the monthly gross profit allows the comparison of the relative performance of each scenario simulated and the identification of the set of bioprocess parameters (final biomass, yield, titer, and productivity) that would lead to the highest possible performance, it alone cannot point if these scenarios represent economically viable projects, in the case of having to build a plant from the ground up rather than working with a pre-existing plant. To determine the viability, a full economic analysis for a 10 year period, with metrics such as the ROI, payback time, NPV and IRR was carried out. Table 9 presents the results of the economic analysis of the growth-associated PHB production simulations with recombinant *E. coli* harboring the NADPH-dependent PHB synthesis pathway under aerobic conditions for the batch process.

**Table 9:** Economic Evaluation of Growth-Associated Production for *E. coli* Harboring the NADPH-Dependent PHB Synthesis Pathway under Aerobic Conditions for the Batch Process

annual net profit	ROI	payback time	net present value (NPV)	internal rate of return (IRR)
kUSD/year	%	years	kUSD	%
–2664	–13	-	–35,961	-
–2478	–12	-	–34,908	-
–1962	–9	-	–31,993	-
–1390	–7	-	–28,763	-
–683	–3	-	–24,768	-
–46	0	-	–21,170	-
683	3	31	–17,050	–16%
1361	7	15	–13,220	–7%
1854	9	11	–10,431	–2%
2132	10	10	–8862	0%
2356	11	9	–7598	2%
2500	12	8	–6785	3%
2499	12	8	–6789	3%
2233	11	9	–8289	1%

It can be seen in Table 9 that for a batch process on the scale of these simulations, not even the scenario that leads to the maximum possible monthly gross profit would result in an economically viable project, in the case of a plant that has to be built from the ground up, followed by an operation period of 10 years, under the conditions tested in the economic analysis. Possibly, the use of fed-batch process, which could increase productivity, could potentially lead to economically viable scenarios. Another possibility is the use of cheaper carbon sources, decreasing therefore the production costs. These possibilities should be explored in future works. The results for the economic analysis of all other growth-associated PHB production simulations and all nongrowth-associated PHB production simulations are available in Supporting Information F and I, respectively.

### Challenges, Limitations, and Prospects

It is worth highlighting that in most of the scenarios simulated, if the limit of PHB content was above 85%, profits would be even higher. This falls in line with the literature where strategies to alter the microorganisms’ morphology to increase the accumulation of PHAs are explored.^64,65^ Something else to address is that while exploration of the trade-off between biomass and product formation and of the possible two-phase production scenarios revealed that *E. coli* has an even higher PHB production potential than *C. necator* thanks to its greater growth rate, in real experiments with these microorganisms without further genetic modifications, *C. necator* usually presents higher yields and accumulation than *E. coli*. One possible explanation for this may be the regulatory effects at play in these microorganisms, meaning that further study of the regulatory system of these microorganisms would help in strategies to improve PHB production in vivo. Another point to address is the limitations of the approach such as the simplification hypotheses behind FBA/DFBA and the use of genome-scale models that do not account for gene regulation, enzyme availability, and physical space limitations and how that may affect fluxes. There is also the difficulty of finding the parameters for uptake kinetics and inhibition constants of certain microorganisms and for certain carbon sources in the literature. In the future, the DySEEP approach could be applied to the production of copolymers; however, some challenges will need to be overcome. Studies on propionate metabolism have contributed to improving the synthesis efficiency of 3HV monomers,^125^ although there is at least one propionate oxidation pathway that has not been elucidated,^126,127^ and therefore, the maximum 3HV yield from propionate has not been reached. Genome-scale metabolic models should consider the role of this additional propionate oxidation pathway, in addition to the well-characterized 2-methylcitrate cycle.^126–129^ In the case of P3HB-*co*-3HHx synthesis, the insertion of PHA biosynthesis genes from *Aeromonas* sp. into a *B. sacchari* mutant deficient in PHA production allowed approximately 50% of the maximum 3HHx yield from hexanoic acid to be achieved.^130^ The deletion of fatty acid β-oxidation components should allow the maximum efficiency of hexanoic acid conversion into 3HHx units to be achieved. But another limitation of the current approach when specifically applied to products like polymers is that FBA/DFBA cannot predict some important properties of the polymers since they are based on stoichiometric modeling. But the results that can be obtained are still very valuable for initial economic assessment. The information obtained with the DySEEP approach provides potential targets to assist metabolic engineering, synthetic biology, and bioreactor operation strategies. The proposed method can be applied to obtain useful information for any bioprocess where there may be a trade-off between biomass and product formation during growth-associated production and any two-phase production processes, provided that data regarding the costs of the main downstream steps as a function of parameters such as final biomass, yield, titer, or productivity is available. The DySEEP approach can also be easily adapted to include any new genome-scale models and any new advances regarding carbon uptake kinetics.

## Conclusions

Expanding upon works where dynamic flux balance analysis is used to assist the pre-evaluation of potential bioprocesses and aid metabolic engineering strategies, this work presents an approach named DySEEP to estimate information such as the scenario that marks the start of positive cash flows and the scenario that leads to the maximum theoretical monthly gross profit for the production of products of interest under different conditions. As a case study, the production of poly-3-hydroxybutyrate was evaluated. Although the DySEEP approach cannot predict important PHB properties such as crystallinity and average molecular weight, since it is based on stoichiometric modeling, the approach can provide potential targets for optimization strategies. Using DFBA simulations with a genome-scale model of *E. coli* with the addition of a NADPH or a NADH PHB synthesis pathway, the monthly gross profit for each simulation that covers the trade-off between growth and product formation was estimated, in the case of growth-associated production. The scenario that leads to the start of positive cash flows and the scenario whose flux distribution leads to the best performance were then identified. For growth-associated PHB production with *E. coli*, it was found that using a NADH-dependent pathway increases the maximum theoretical monthly gross profit by 4% in comparison to using a NADPH-dependent pathway under aerobic conditions and allowed a positive cash flow to be achievable under anaerobic conditions. The best performance with a NADH-dependent pathway is from a scenario that achieves a yield of 0.50 g PHB/g glc with its respective titer of 12.60 g PHB/L and productivity of 0.88 g PHB/L h during aerobic batch culture, leading to a monthly gross profit of 101,000 USD. Even though the NADH-dependent pathway greatly increased the PHB production potential for *E. coli* under anaerobiosis, the performance is still below the aerobic options in the conditions simulated. This means that the savings with energy and aeration costs were not enough to compensate for the lower growth rate and yield.

With the aid of IMFLer, the flux distributions of the central metabolism for the simulated scenario that leads to the best performance along with the flux distributions of a simulation constrained with experimental data from the literature were drawn on a metabolic map and compared. This, together with FVA, allowed the suggestion of genetic modifications that could potentially push the internal flux distribution of the cells from real experimental scenarios closer to that of the ideal flux distribution identified in the growth-associated simulations. Some of the genetic modifications include interruption of the oxidative phase of the pentose phosphate pathway and of acetate excretion, interruption of the reaction catalyzed by the 2-oxoglutarate dehydrogenase enzyme or downregulation of the TCA cycle, and overexpression of the PntAB transhydrogenase, modifications that are aligned with works where an increase of the PHB yield and content was successfully achieved.

Two-phase PHB production with recombinant *C. necator*, *E. coli,* and *S. cerevisiae* was also investigated. In this scenario, a question of how much of a given total amount of available glucose is allocated to the growth phase and how much to the production phase in order to maximize profit arises or, in other words, when the shift from growth to PHB phase should occur. The best scenario for the two-phase simulations for both *E. coli* and *C. necator* is achieved using 20% (mol) in the growth phase and the remaining 80% (mol) in the production phase, leading to a monthly gross profit of 108,000 and 59,000 USD, respectively. The finding that a somewhat early shift from growth to PHB production as long as flux is effectively directed to PHB in the production phase leads to the best performance is aligned with a few works in which genetic toggle-switches were tested experimentally to improve PHB production. Meanwhile, the simulations revealed that no positive cash flow was achievable with *S. cerevisiae* in the scale and conditions tested. It was shown, therefore, that the DySEEP approach is useful to give insight into important bioprocess parameters to assist in the pre-evaluation of potential bioprocesses under different scenarios and also to set potential targets for metabolic engineering and synthetic biology strategies.

As for future work, possibilities such as building a MATLAB program that allows implementation of the DySEEP approach in fed-batch processes and then evaluates the effect of the use of different carbon sources will be explored.

## Supplementary Material



## Notes

Full program together with instructions on how to use it, scripts for adding the PHB synthesis pathway in the *E. coli* and *S. cerevisiae* models and glucose transport in the *C. necator* model, detailed description of all the steps used to estimate the costs of upstream, bioreactor operation, and downstream, complete tables with the full economic analysis for each condition, all the metabolic map figures, and the genome-scale models and MATLAB code files (ZIP)
