# Supplementary material for: Analysis of Poly-3-Hydroxybutyrate Production with Different Microorganisms Using the Dynamic Simulations for Evaluation of Economic Potential Approach
Source: ACS Omega. 2025 Jun 11;10(26):27756–74. doi: 10.1021/acsomega.4c11178 (PMC12242656; doi:10.1021/acsomega.4c11178)
Supplement: Supplementary file 1 [file ao4c11178_si_001.zip › Supporting Information/Supporting Information D/bioreactor operation cost for PHB phase under aerobic conditions.docx]

Supplementary material D – Calculation of operational costs for aerobic simulations during PHB phase

The following algorithm is used to calculate the costs with aeration, agitation and
cooling for the PHB production phase of each two-phase production simulations, under
aerobic conditions, following the procedure and equations described in the supplementary material C. In order to use this algorithm, first choose how much of the total glucose will be used for each phase, and enter the chosen glucose concentration (So) for the PHB phase in this algorithm. Run the growth phase of the two-phase production DFBA simulation in MATLAB using the provided program, and find the final biomass concentration (concentration_X) and enter the value on this algorithm. During the PHB production phase there will be no biomass formation and the biomass concentration will stay the same as the final biomass concentration from the growth phase simulation. Run the PHB phase DFBA simulation (PHB as objective function) in MATLAB using the provided program. Then, use standard FBA, for instance the Escher-FBA application, to find the maximum theoretical product yield (ymax) and the maximum flux to PHB synthesis possible (vPHB_max), given the uptake rate of glucose and oxygen used, and enter the appropriate values in this algorithm. Also enter the maximum oxygen uptake for the microorganism (vO2_max). Copy this algorithm and paste it in MATLAB’s command window and the final titer (Tfinal), the time to reach the final titer (t_OP), and the aeration, agitation and cooling costs will be calculated.

% Bioreactor dimensions:

% Height of the bioreactor = 15 m
% Height of medium in the bioreactor = 10.19 m
% Bioreactor diameter = 5 m % Bioreactor impeller diameter = 2.25 m
% Bioreactor area = 19.63 m^2^
% Volume of medium in the bioreactor = 200000 L = 200 m^3^

% Medium properties and operational conditions:
% Mineral medium estimated density = 1032 kg/ m^3^
% Hydrostatic pressure in the bottom of the bioreactor = 1032 * 9.81*10.19 = 103162.74 pa = 1.02 atm
% Absolute pressure in the bottom of the bioreactor (Preact) = 263200 pa = 2.60 atm

% Volume of medium in the bioreactor: volume = 200000; % L

% Aeration costs:

concentration_X = “enter biomass concentration, which for the PHB production phase in the two-phase PHB production simulations is a constant”;
vO2_max = “enter maximum oxygen uptake rate for the microorganism being simulated”;
% Oxygen uptake rate OUR mmolO2/L.h:
OUR_t = vO2_max * concentration_X ;
% oxygen molar rate mols O2/s
nO2_t = ((((OUR_t * 10000)./1000)./3600)./0.20);
% O2 flow rate m^3^/s:
QO2_in_t = ((nO2_t *0.082* 298.15)./1)./1000;
% Air flow rate m^3^/s in the inlet:
Qair_in_t = QO2_in_t./0.21;
% Air flow rate m^3^/s in the outlet:
Qair_out_t = ((1* Qair_in_t)./298.15)*(310.15/2.60);
% Power consumption of the compressor (PC) kW:
PC_t = ((101325* Qair_in_t)*(1.4/(1.4-1))*(((263200/101325)((1.4-1)/1.4)) - 1)*(1/0.7)./1000);
% Energy consumption of the compressor (kWh) for each time step:
So = “enter the concentration of glucose allocated to the PHB production phase for the two-phase PHB production simulation (mol glu/L)”;
ymax = “enter the maximum theoretical PHB yield with FBA for the condition simulated (molPHB/mol glu)”;
vPHB_max = “enter the maximum theoretical flux to PHB possible with FBA given the chosen glucose uptake, for the condition simulated (mmol PHB/CDW h)”;
Tfinal = ymax*So; % mol PHB/L
t_OP = (Tfinal)/((vPHB_max/1000)*concentration_X); % h
ECtotal = PC_t * t_OP;
Cost_aera = 0.126* ECtotal;

% Cost of agitation:

% Concentration of O2 in the medium is constant at a chosen value of 3.2 mg/L = 0.1 mmol/L
% Concentration of O2 at saturation for 37 ◦C is = 6.71 mg/L = 0.21 mmol/L
kLa_t = ((OUR_t./( 0.21 - 0.1))./3600); % kLa in s-1
Vsuper_t = ((Qair_out_t)./19.63); % superficial gas velocity in m/s:
PS_t = (((kLa_t*((volume./1000)^0.7))./(0.002 *((Vsuper_t).^0.2)))).^(10/7); % Gassed power input in W
% Energy consumption of the stirrer (kWh) for each time step:
EStotal = (PS_t./1000)*t_OP;
Cost_agi = EStotal*0.126;

% Cost of cooling:

Qheat_t = (0.50*OUR_t *10000)./3600; % Qheat em kW
% Energy consumption of the cooling system (kWh) for each time step:
EMtotal = Qhea_t*t_OP;
Cost_cool = (0.126/0.7)*(EStotal + EMtotal);

% Print results:
Cost_aera
Cost_agi
Cost_cool
