# Supplementary material for: Analysis of Poly-3-Hydroxybutyrate Production with Different Microorganisms Using the Dynamic Simulations for Evaluation of Economic Potential Approach
Source: ACS Omega. 2025 Jun 11;10(26):27756–74. doi: 10.1021/acsomega.4c11178 (PMC12242656; doi:10.1021/acsomega.4c11178)
Supplement: Supplementary file 1 [file ao4c11178_si_001.zip › Supporting Information/Supporting Information D/bioreactor operation cost for PHB phase under anaerobic conditions.docx]

Supplementary material D – Calculation of operational costs for anaerobic simulations during PHB phase

The following algorithm is used to calculate the costs with agitation and cooling for
the PHB production phase of each two-phase production simulations, under anaerobic
conditions, following the procedure and equations described in the supplementary material C. In order to use this algorithm, first choose how much of the total glucose will be used for each phase, and enter the chosen glucose concentration (So) for the PHB phase in this algorithm. Run the growth phase of the two-phase production DFBA simulation in MATLAB using the provided program, and find the final biomass concentration (concentration_X) and enter the value on this algorithm. During the PHB production phase there will be no biomass formation and the biomass concentration will stay the same as the final biomass concentration from the growth phase simulation. Run the PHB phase DFBA simulation (PHB as objective function) in MATLAB using the provided program. Then, use standard FBA, for instance the Escher-FBA application, to find the maximum theoretical product yield (ymax) and the maximum flux to PHB synthesis possible (vPHB_max), given the uptake rate of glucose used, and enter the appropriate values in this algorithm. Copy this algorithm and paste it in MATLAB’s command window and the final titer (Tfinal), the time to reach the final titer (t_OP), and the agitation and cooling costs will be calculated.

% Bioreactor dimensions:

% Height of the bioreactor = 15 m
% Height of medium in the bioreactor = 10.19 m
% Bioreactor diameter = 5 m % Bioreactor impeller diameter = 2.25 m
% Bioreactor area = 19.63 m2
% Volume of medium in the bioreactor = 200000 L = 200 m3

% Medium properties and operational conditions:
% Mineral medium estimated density = 1032 kg/ m3

% Hydrostatic pressure in the bottom of the bioreactor = 1032 * 9.81*10.19 = 103162.74 pa = 1.02 atm
% Absolute pressure in the bottom of the bioreactor (Preact) = 263200 pa = 2.60 atm

% Volume of medium in the bioreactor: volume = 200000; % L

% Aeration costs:

% Zero, as there is no aeration for the anaerobic cultures.

% Cost of agitation:

concentration_X = “enter biomass concentration, which for the PHB production phase in the two-phase PHB production simulations is a constant”;
So = “enter the concentration of glucose allocated to the PHB production phase for the two-phase PHB production simulation (mol glu/L)”;
ymax = “enter the maximum theoretical PHB yield with FBA for the condition simulated (molPHB/mol glu)”;
VPHB_max = “check the maximum theoretical flux to PHB possible with FBA given the chosen glucose uptake, for the condition simulated (mmol PHB/CDW h)”;
Tfinal = ymax*So; % mol PHB/L
t_OP = (Tfinal)/((VPHB_max/1000)*concentration_X); % h
% Agitation of 50 rpm was assumed. With this agitation and the medium properties being similar to those of water, the Reynolds number (Re) calculated is 4336335, therefore, a turbulent flow. Choosing a flat-blade impeller (W/D=1/5), the power number (c) is then 4. With that, the power for stirring can be calculated:
% Power = (4)*(1032 kg/m3)*(0.83 s−1)^3*(2.25 m)^5
% Power = 136108.9 W
% Power = 136.1 kW
% With the stirring power needed, the energy for stirring is calculated by multiplying it by the duration time of the growth phase (t_OP) from the simulation.
EStotal = 136.1*t_OP;
Cost_agi = EStotal*0.126;

% Cost of cooling:

EMtotal = (So*volume*235)/3600; % kWh
Cost_cool = (0.126/0.7)*(EStotal + EMtotal);

% Print results:
Cost_agi
Cost_cool
