# Supplementary material for: Analysis of Poly-3-Hydroxybutyrate Production with Different Microorganisms Using the Dynamic Simulations for Evaluation of Economic Potential Approach
Source: ACS Omega. 2025 Jun 11;10(26):27756–74. doi: 10.1021/acsomega.4c11178 (PMC12242656; doi:10.1021/acsomega.4c11178)
Supplement: Supplementary file 1 [file ao4c11178_si_001.zip › Supporting Information/Supporting Information D/bioreactor operation cost for growth-associated or growth phase simulations under aerobic conditions.docx]

Supplementary material D – Calculation of operational costs for aerobic simulations during growth

The following algorithm is used to calculate the costs with aeration, agitation and
cooling for each simulation with a flux distribution that explores the trade-off between
biomass and product formation on the growth-associated production simulations, or the growth phase of the two-phase production simulations, under aerobic conditions, following the procedure and equations described in the supplementary material C. In order to use this algorithm, first run the desired DFBA simulation in MATLAB using the provided program. Check the index in the time vector for the moment where the end of the exponential phase is reached and enter the appropriate value in the “final_time_index” variable in the following algorithm. With that, copy this algorithm and paste it in MATLAB’s command window and the aeration, agitation and cooling costs will be calculated.

% Bioreactor dimensions:

% Height of the bioreactor = 15 m
% Height of medium in the bioreactor = 10.19 m
% Bioreactor diameter = 5 m % Bioreactor impeller diameter = 2.25 m
% Bioreactor area = 19.63 m^2^
% Volume of medium in the bioreactor = 200000 L = 200 m^3^

% Medium properties and operational conditions:
% Mineral medium estimated density = 1032 kg/ m^3^

% Hydrostatic pressure in the bottom of the bioreactor = 1032 * 9.81*10.19 = 103162.74 pa = 1.02 atm
% Absolute pressure in the bottom of the bioreactor (Preact) = 263200 pa = 2.60 atm

% Biomass concentration vector: concentration_X = y(:,1);

% Volume of medium in the bioreactor: volume = 200000; % L time_step =0.05; % Step size used in this study, but user can change it if desired. final_time_index = “enter the index from the time vector for the moment where the end of the exponential is reached, in the simulation”

% Aeration costs:

% Oxygen uptake rate OUR mmolO2/L.h:

VO2_max = “maximum oxygen uptake rate for the microorganism being simulated”;

OUR_t = VO2_max * concentration_X

% Oxygen molar rate molO2/s that needs to be delivered to the bioreactor. Assuming a transfer of 20% of the oxygen mols from the bubble to the medium, the following calculations were made.
nO2_t = ((((OUR_t .* volume)./1000)./3600)./0.20);

% O2 flow rate m^3^/s

QO2_in_t = ((nO2_t *0.082* 298.15)./1)./1000;

% Air flow rate m^3^/s in the inlet

Qair_in_t = QO2_in_t./0.21;

% Air flow rate m^3^/s in the outlet

Qair_out_t = ((1* Qair_in_t)./298.15)*(310.15/2.60);

% Power consumption of the compressor (pc) kW:

PC_t = ((101325* Qair_in_t )*(1.4/(1.4-1))*(((263200/101325)^((1.4-1)/1.4)) - 1)*(1/0.7)./1000);

% Energy consumption of the compressor (kWh) for each time step:
EC_t = PC_t* time_step;
% Total energy consumption of the compressor (EC = ∑PC.∆t) kWh:
EC_seg = EC_t(1:final_time_index); ECtotal = sum(EC_seg);
Cost_aera = 0.126* ECtotal;

% Cost of agitation

% Concentration of O2 in the medium is constant at a chosen value of 3.2 mg/L = 0.1 mmol/L
% Concentration of O2 at saturation for 37 ◦C is = 6.71 mg/L = 0.21 mmol/L
kLa_t = ((OUR_t./( 0.21 - 0.1))./3600); % kLa in s-1
Vsuper_t = ((Qair_out_t)./19.63); % Superficial gas velocity in m/s

PS_t = (((kLa_t*((volume./1000)^0.7))./(0.002 *((Vsuper_t).^0.2)))).^(10/7); % Gassed power input in W

% Energy consumption of the stirrer (kWh) for each time step:

ES_t = (PS_t./1000)*time_step;
% Total energy consumption of the stirrer (ES = ∑PS.∆t) kWh:
ES_seg = ES_t(1: final_time_index);.
EStotal = sum(ES_seg);
Cost_agi = EStotal*0.126;

% Cost of cooling:
Qheat_t = (0.50*OUR_t*10000)./3600; % Qheat in kW:
% Energy consumption of the cooling system (kWh) for each time step:
EM_t = Qheat_t* time_step;
% Total energy consumption of the cooling system (EM = ∑Qheat.∆t) kWh:
EM_seg = EM_t(1: final_time_index); EMtotal = sum(EM_seg); Cost_cool = (0.126/0.7)*(EStotal + EMtotal);

% Print results:
Cost_aera
Cost_agi
Cost_cool
