# Supplementary material for: Analysis of Poly-3-Hydroxybutyrate Production with Different Microorganisms Using the Dynamic Simulations for Evaluation of Economic Potential Approach
Source: ACS Omega. 2025 Jun 11;10(26):27756–74. doi: 10.1021/acsomega.4c11178 (PMC12242656; doi:10.1021/acsomega.4c11178)
Supplement: Supplementary file 1 [file ao4c11178_si_001.zip › Supporting Information/Supporting Information D/bioreactor operation cost for growth-associated or growth phase under anaerobic conditions.docx]

Supplementary material D – Calculation of operational costs for anaerobic simulations during growth

The following algorithm is used to calculate the costs with aeration, agitation and
cooling for each simulation with a flux distribution that explores the trade-off between
biomass and product formation on the growth-associated production simulations, or the growth phase of the two-phase production simulations, under anaerobic conditions, following the procedure and equations described in the supplementary material C. In order to use this algorithm, first run the desired DFBA simulation in MATLAB using the provided program. Check the initial glucose concentration and the duration of the growth phase and enter the appropriate values in the “So” and the “t_OP” variables in the following algorithm. With that, copy this algorithm and paste it in MATLAB’s command window and the aeration, agitation and cooling costs will be calculated.

% Bioreactor dimensions:

% Height of the bioreactor = 15 m
% Height of medium in the bioreactor = 10.19 m
% Bioreactor diameter = 5 m % Bioreactor impeller diameter = 2.25 m
% Bioreactor area = 19.63 m^2^
% Volume of medium in the bioreactor = 200000 L = 200 m^3^

% Medium properties and operational conditions:
% Mineral medium estimated density = 1032 kg/ m^3^

% Hydrostatic pressure in the bottom of the bioreactor = 1032 * 9.81*10.19 = 103162.74 pa = 1.02 atm
% Absolute pressure in the bottom of the bioreactor (Preact) = 263200 pa = 2.60 atm

% Volume of medium in the bioreactor: volume = 200000; % L

% Aeration costs:

% Zero, as there is no aeration for the anaerobic cultures.

% Agitation costs:

% Agitation of 50 rpm was assumed. With this agitation and the medium properties being similar to those of water, the Reynolds number (Re) calculated is 4336335, therefore, a turbulent flow. Choosing a flat-blade impeller (W/D=1/5), the power number (c) is then 4. With that, the power for stirring can be calculated:
% Power = (4)*(1032 kg/m^3^)*(0.83 s−1)^3*(2.25 m)^5
% Power = 136108.9 W
% Power = 136.1 kW

% With the stirring power needed, the energy for stirring is calculated by multiplying it by the duration time of the growth phase (t_OP) from the simulation.

t_OP = “ enter the duration of the growth phase from the simulation in hours”
EStotal = 136.1*t_OP;
Cost_agi = EStotal*0.126;

% Cost of cooling:

So = “enter the initial glucose concentration in mmol/L” glucose_consumed = (So*volume)/1000; % mols EMtotal = (glucose_consumed*235)/3600; % kWh
Cost_cool = (0.126/0.7)*(EStotal + EMtotal);

% Print results:
Cost_agi
Cost_cool
