# Supplementary material for: Analysis of Poly-3-Hydroxybutyrate Production with Different Microorganisms Using the Dynamic Simulations for Evaluation of Economic Potential Approach
Source: ACS Omega. 2025 Jun 11;10(26):27756–74. doi: 10.1021/acsomega.4c11178 (PMC12242656; doi:10.1021/acsomega.4c11178)
Supplement: Supplementary file 1 [file ao4c11178_si_001.zip › Supporting Information/Supporting Information summary.pdf]

Analysis of poly-3-hydroxybutyrate production with different microorganisms using the Dynamic Simulations for Evaluation of Economic Potential (DySEEP) approach

Willians O. Santos,[†] Rafael D. de Oliveira,[‡] José G. C. Gomez,[¶] and Galo A. C. Le Roux[*,†]

[†]*Department of Chemical Engineering Polytechnic School, University of São Paulo. Av. Prof. Lineu Prestes, 580, postcode 05508-220, São Paulo, Brazil*

[‡]*Department of Chemical Engineering, Norwegian University of Science and Technology (NTNU). Torgarden, NO-7491, postcode 8900, Trondheim, Norway*

[¶]*Institute of Biomedical Sciences, Bioproducts laboratory, University of São Paulo. Av. Prof. Lineu Prestes, 2415, postcode 05508-000, São Paulo, Brazil*

E-mail: galoroux@usp.br

Supporting Information summary

Supporting Information A - The full program together with instructions on how to use it.

Supporting Information B - Scripts for adding the PHB synthesis pathway in the *E. coli* and *S. cerevisiae* models, and glucose transport in *C. necator* model.

Supporting Information C - The detailed description of all the steps used to estimate the costs of upstream, bioreactor operation, and downstream.

Supporting Information D - MATLAB scripts to calculate the bioreactor operation costs using the described equations and the parameters obtained with each simulation.

Supporting Information E - Tables with the equipment costs and multipliers used to estimate the total investment.

Supporting Information F - The full tables for all growth-associated simulations are available.

Supporting Information G -Versions with a zoom-in/out feature for all the figures of metabolic maps generated for this study can be seen.

Supporting Information H - The Tables with the results of the FVA simulations for the other growth associated simulation scenarios are available.

Supporting Information I - The full tables for all nongrowth-associated simulations are available.
