# Supplementary material for: Analysis of Poly-3-Hydroxybutyrate Production with Different Microorganisms Using the Dynamic Simulations for Evaluation of Economic Potential Approach
Source: ACS Omega. 2025 Jun 11;10(26):27756–74. doi: 10.1021/acsomega.4c11178 (PMC12242656; doi:10.1021/acsomega.4c11178)
Supplement: Supplementary file 1 [file ao4c11178_si_001.zip › Supporting Information/Supporting Information B/adding PHB synthesis pathway.docx]

Supplementary material A - code for addition of PHB synthesis pathway in *E. coli* and *S. cerevisiae* models and glucose transport to *C. necator* model

The following lines of code were used in COBRA toolbox for MATLAB, to add a NADPH-dependent or a NADH-dependent PHB synthesis pathway to the *E. coli* and *S. cerevisiae* models (Adapted from Heshiki 2013), and the glucose transport to the *C. necator* model.

For *Escherichia coli* iML1515:

% Add metabolites
model = addMultipleMetabolites(model,’3hbcoa__R_c’,’3hb_c’,’3hb_p’,’3hb_e’,’metNames’,’(R)-
3-hydroxybutyryl-CoA’,’3-hydroxybutyrate’, ’3-hydroxybutyrate’,’3-hydroxybutyrate’,’metCharges’,
[ -4 -4 -4 -4], ’metFormulas’, ’C25H42N7O18P3S’,’C4H6O2’, ’C4H6O2’,’C4H6O2’);

% Add Reaction (beta-ketothiolase)
model = addReaction(model,’ACACT1r’, ’2 accoa_c <=> aacoa_c + coa_c’);

% Add Reaction (acetoacetyl-CoA reductase):

% Add Reaction (acetoacetyl-CoA reductase NADPH dependent)

model = addReaction(model,’AACOAr’, ’aacoa_c + nadph_c + h_c <=> 3hbcoa__R_c +
nadp_c’);

or

% Add Reaction (acetoacetyl-CoA reductase NADH dependent)
model = addReaction(model,’AACOAr’, ’aacoa_c + nadh_c + h_c <=> 3hbcoa__R_c +
nad_c’);

% Add Reaction (PHB polymerase)
model = addReaction(model,’3HB’, ’3hbcoa__R_c -> 3hb_c + coa_c’);

% Add Reactions (PHB diffusion)
model = addReaction(model,’3HBtpp’,’3hb_c <=> 3hb_p’);
model = addReaction(model,’3HBtex’,’3hb_p <=> 3hb_e’);
model = addReaction(model,’EX_3hb_e’,’3hb_e <=>’);

For *Saccharomyces cerevisiae* iMM904:

% Add metabolites

model = addMultipleMetabolites(model,{'3hbcoa__R_c','3hb_c','3hb_e'},'metNames',{'(R)-3-hydroxybutyryl-CoA','3-hydroxybutyrate','3-hydroxybutyrate'},'metCharges', [ -4 -4 -4], 'metFormulas', {'C25H42N7O18P3S','C4H6O2','C4H6O2'});

% Add Reaction (acetoacetyl-CoA reductase ):

% Add Reaction (acetoacetyl-CoA reductase NADPH dependent)

model = addReaction(model,'AACOAr', 'aacoa_c + nadph_c + h_c <=> 3hbcoa__R_c + nadp_c');

or

% Add Reaction (acetoacetyl-CoA reductase NADH dependent)

model = addReaction(model,'AACOAr', 'aacoa_c + nadh_c + h_c <=> 3hbcoa__R_c + nad_c');

% Add Reaction (PHB polymerase)

model = addReaction(model,'3HB', '3hbcoa__R_c -> 3hb_c + coa_c');

% Add Reactions (PHB diffusion)

model = addReaction(model,'3HBtex','3hb_c <=> 3hb_e');

model = addReaction(model,'EX_3hb_e','3hb_e <=>');

For *Cupriavidus necator* RehMBEL1391:

% Add metabolites

model = addMultipleMetabolites(model,{ 'gluco[e]','gluco[c]'},'metNames',{' D-Glucose',' D-Glucose'},'metCharges', [ 0, 0], 'metFormulas', { 'C6H12O6','C6H12O6'});

% Add Reaction (glucose uptake)

model = addReaction(model,'EX_gluco_e', 'gluco[e] <=>');

% Add Reaction (D-glucose transport via ABC system)

model = addReaction(model,'GLUCOabc', 'gluco[e] + atp[c] + h2o[c] -> pi[c] + gluco[c] + adp[c] + h[c] ');

% Add Reaction (Hexokinase (D-glucose: ATP))

model = addReaction(model,'HEXglucose', 'gluco[c] + atp[c] -> adp[c] + g6p[c] + h[c]');
