# Supplementary material for: Analysis of Poly-3-Hydroxybutyrate Production with Different Microorganisms Using the Dynamic Simulations for Evaluation of Economic Potential Approach
Source: ACS Omega. 2025 Jun 11;10(26):27756–74. doi: 10.1021/acsomega.4c11178 (PMC12242656; doi:10.1021/acsomega.4c11178)
Supplement: Supplementary file 1 [file ao4c11178_si_001.zip › Supporting Information/Supporting Information A/Simple Instructions in how to perform the DFBA simulations.docx]

**Simple Instructions in how to perform the DFBA simulations**

In order to run a DFBA simulation, first, open the files simulation.m, FBA.m and DFBA.m. On the simulation.m file, enter the initial concentrations of biomass (g/L), glucose (mmol/L), ammonia (mmol/L), PHB (mmol/L), acetate (mmol/L), lactate (mmol/L), ethanol (mmol/L), formate (mmol/L), and succinate (mmol/L), respectively. It is also possible to remove or add new metabolites to be tracked by the simulations, but that will require changes in other sections of the scripts as well. The sections that would require changes in this instance are marked in the m.files. Next, enter the total time duration of the simulation and the time step (in hours). The total duration can be any amount of time that is enough for all glucose to be consumed.

Now, go to the FBA.m file. Uncomment the indexes and load the model of the microorganism that will be used in the simulation. For growth associated PHB production simulations that explore the trade-off between biomass and PHB formation, keep the biomass objective function (default), and on the constrains for the linprog function section, fix the flux to PHB synthesis to any desired number, within the limits of the maximum PHB yield of the chosen microorganism. For non growth-associated PHB production simulations, change the objective function to PHB synthesis. For that, just uncomment the block of code that changes the objective function, as marked in the FBA.m file itself.

Go to the DFBA.m file. Uncomment the oxygen and the glucose uptake expressions for the model of the microorganism that will be used in the simulation. Finally, go back to the simulation.m file and run. Running the simulation.m file will create a concentrations matrix (y) and a time vector (t) variable, and a plot showing the biomass and metabolites as a function of time is created. With that, the final biomass, final yield, final titer, and the time to reach the final titer (and hence, the productivity) can be identified.
