# Supplementary material for: Analysis of Poly-3-Hydroxybutyrate Production with Different Microorganisms Using the Dynamic Simulations for Evaluation of Economic Potential Approach
Source: ACS Omega. 2025 Jun 11;10(26):27756–74. doi: 10.1021/acsomega.4c11178 (PMC12242656; doi:10.1021/acsomega.4c11178)
Supplement: Supplementary file 1 [file ao4c11178_si_001.zip › Supporting Information/Supporting Information C/estimation of monthly gross profit.pdf]

Analysis of poly-3-hydroxybutyrate production with different microorganisms using the Dynamic Simulations for Evaluation of Economic Potential (DySEEP) approach

Willians O. Santos,† Rafael D. de Oliveira,‡ José G. C. Gomez,¶ and Galo A. C. Le Roux*,†

†*Department of Chemical Engineering Polytechnic School, University of São Paulo. Av. Prof. Lineu Prestes, 580, postcode 05508-220, São Paulo, Brazil*

‡*Department of Chemical Engineering, Norwegian University of Science and Technology (NTNU). Torgarden, NO-7491, postcode 8900, Trondheim, Norway*

¶*Institute of Biomedical Sciences, Bioproducts laboratory, University of São Paulo. Av. Prof. Lineu Prestes, 2415, postcode 05508-000, São Paulo, Brazil*

E-mail: galoroux@usp.br

1 Material and Methods

1.1 Determining the monthly gross profit

There are dedicated software for economical evaluation of bioprocesses such as SuperPro Designer^{TM}, but they are not free software (Petrides, 2013). The gross profit of a specific bioprocess however can be roughly estimated with relations found in the literature expressed as functions of the process parameters. The proposed metric to evaluate the simulated cells from the growth associated PHB production simulations and the two-phase PHB production simulations is the monthly gross profit. This work presents a relative straightforward method to estimated the monthly gross profit using design equations found in the literature, together with the parameters obtained in the simulations. Essentially, the monthly gross profit can be expressed as a function of the final biomass, yield, titer, and productivity, obtained from each simulation. The book by Clarke (2013) points out Equation (1), where the gross profit of a process can be calculated.

$$GP = R - (RM + OP + US + DS) \tag{1}$$

Where GP is the gross profit, R is the revenue obtained from the product sales, RM is the raw material cost, OP is the reactor operational cost, US is the upstream expenses and DS is the downstream expenses. The revenue per batch obtained from product sales can be calculated as shown in Equation (2).

$$R = T\ V\ \$P \tag{2}$$

Where T is the titer, that is, the final product concentration (kg/L), V is the bioreactor volume (L) and $\$P$ is the product selling price (USD/kg). It can be seen that the revenue obtained increases linearly with an increase in titer. The selling price of PHB used in the simulation was USD 5.5/kg (Pavan et al., 2019) and the usual PHB recovery is of 95% (Harding, Dennis, Vonblottnitz, & Harrison, 2007). The raw material cost per batch is calculated according to Equation (3).

$$RM = S_o\ V\ \$S \tag{3}$$

Where S_o is the initial substrate concentration (kg/L), V is the reactor volume, and $\$S$ is the substrate purchase price (USD/kg). The price of glucose is USD 0.35/kg (Petrides, 2013). So for the simulations in case, the chosen initial glucose concentration of 25 g/L and the volume of 200 m^3 leads to a raw material cost of USD 1750.

The upstream cost was simplified as the cost of medium and sterilization, and since the type and volume of medium used in all simulations is the same and not a function of yield, titer and productivity, the upstream cost was treated as a constant. The work by Cardoso et al. (2020) provides a table with the prices for many compounds used in bacterial culture media. Assuming a mineral medium with the composition as presented in Table 1, the cost of the 200 m^3 medium used for the simulations is roughly USD 1942.4.

Calculation of sterilization cost was done by estimating how much steam would be needed to heat the medium from 25 °C to 121 °C, usual temperature used for sterilization (MERCK, 2000). Assuming the specific heat capacity of the medium as being the same as of water, and using thermodynamic tables available in the book by Van Ness, Smith, and Abbott (2000), the average specific heat capacity for the medium in this temperature range was calculated as 4.205 kJ/kg.K . The density of the medium was estimated as being 1032 kg/m^3, so the volume of 200 m^3 weights 206,400 kg. This implies that a heat transfer of 83,319,552 kJ would be necessary to

Table 1: Mineral medium composition

Compound	Concentration
Na_2HPO_4	3.5 g/L
KH_2PO_4	1.5 g/L
$(NH_4)_2SO_4$	1.0 g/L
citr. $Fe.NH_4$	0.06 g/L
$MgSO_4.7H_2O$	0.20 g/L
$CaCl_2.2H_2O$	0.01 g/L
Kanamycin	0.05 g/L

Source: Ramsay et al. (1990)

heat the medium from 25 °C to 121 °C. Steam at 4 bar(g) is usually applied for heating, and in this condition the steam specific enthalpy of evaporation is 2108.1 kJ/kg (SPIRAX SARCO, 2021), meaning that a mass of steam of 39523.53 kg would be required. The cost of steam is USD 0.004/kg (Pavan et al., 2019), so the cost to sterilize the 200 m^3 medium is USD 158.1. Summing up the medium and sterilization cost gives a upstream cost of USD 2100.5.

In simple terms, the reactor operational costs can be broken down as the aeration costs (in the case of aerobic process), agitation cost, and cooling costs. The work by Cardoso et al. (2020) presents a methodology to estimate the operational costs of bioprocesses with a few design equations. These equations were adapted to the data available and information that can be retrieved when running FBA/DFBA simulations. The aeration costs are mainly related with the compressor power consumption. The oxygen demand changes throughout the culture as the biomass increases, so in order to keep a constant oxygen concentration in the medium, the aeration has to increase accordingly. In this work, it is assumed that the oxygen concentration is kept at 0.1 mmol/L, which is a realistic value (Hanly, Urello, & Henson, 2012). The oxygen uptake rate can be calculated with Equation (4).

$$OUR(t) = V_{O_2,max}\, X(t) \tag{4}$$

Where $OUR(t)$ is the oxygen uptake rate (mmol O_2/L.h) at any given time and $X(t)$ is the biomass concentration at any given time (g/L). Chapter seven from the book Industrial Waste Treatment Handbook points that aeration systems commonly have a low oxygen transfer efficiency, where usually up to around 15% of the oxygen supplied is actually used by the microorganism. Adopting a oxygen transfer efficiency of 20%, the oxygen flow that has to be delivered to the bioreactor is given by Equation (5).

$$n_{O_2}(t) = \frac{OUR(t)\, V}{100\ 3600\ 0.20} \tag{5}$$

Where $n_{O_2}(t)$ is the oxygen flow rate at any given time (mols/s). Using Clapeyron's equation of ideal gas (Dincer & Zamfirescu, 2018) for the inlet conditions, the oxygen volumetric flow rate that needs to be delivered to the bioreactor is presented in Equation (6).

$$Q_{O_2,in}(t) = \frac{n_{O_2}(t)\, R\, T_{in}}{P_{in}\ 1000} \tag{6}$$

Where $Q_{O_2,in}(t)$ is the oxygen volumetric flow rate at any given time (m^3/s), R is the ideal gas constant (0.082 atm.L/mol.K), T_{in} is the air temperature in the inlet (298.15 K) and P_{in} is the pressure in the inlet (1 atm). And knowing that the air composition is 21%(vol) oxygen, the air flow rate that has to be given to the bioreactor in order to deliver the required oxygen amount is given by Equation (7).

$$Q_{air,in}(t) = \frac{Q_{O_2,in}(t)}{0.21} \tag{7}$$

Where $Q_{air,in}(t)$ is the inlet air flow rate at any given time (m^3/s). For a bioreactor of 295 m^3 with a height to diameter ratio of 3 (Mediboyina et al., 2022), and hence, a height of 15 m and diameter of 5 m, the height of the medium in the bioreactor, given it´s volume of 200 m^3 was of 10.2 m. Given the medium density of 1032 kg/m^3, the static pressure in the bioreactor is of 103162.74 pa (1.02 atm). Bioreactor with *E. coli* can work with a pressure of around 1.6 bar (1.58 atm) (Lopes, Belo, & Mota, 2014). Therefore, the absolute pressure in the bioreactor is 263200 pa (2.60 atm). With the inlet air flow rate and applying Clapeyron's equation (Dincer & Zamfirescu, 2018) for the bioreactor conditions, the air flow rate in the outlet is calculated, as shown in Equation (8).

$$Q_{air,out}(t) = \frac{P_{in}\ Q_{air,in}(t)}{T_{in}} \frac{T_{out}}{P_{out}} \tag{8}$$

Where $Q_{air,out}(t)$ is the outlet air flow rate at any given time (m^3/s), T_{out} is the outlet air temperature (310.15 K), and P_{out} is the pressure in the outlet (2.60 atm). The compressor power consumption is given by Equation (9).

$$P_C(t) = \frac{P_{in}\ Q_{air,in}(t)}{n_c} \frac{\gamma}{\gamma - 1} \left(\frac{P_{reac}}{P_{in}}\right)^{\left(\left(\frac{\gamma-1}{\gamma}\right)-1\right)} \frac{1}{1000} \tag{9}$$

Where $P_C(t)$ is the compressor power consumption (kW) at any given time, P_{in} is the inlet pressure, which is the ambient pressure of 101325 pa, P_{reac} is the reactor pressure of 263200 pa, γ is the isoentropic exponent (1.4) and n_c is the efficiency number of the compressor (0.7). With the compressor power consumption for each time step of the simulation determined, the energy consumption of the compressor can be calculated using Equation (10).

$$E_C = \sum P_C\ \Delta t \tag{10}$$

Where E_C is the compressor total energy consumption (kWh) and when using MATLAB and DFBA, the Δt is the time step of the simulation and P_C the compressor power consumption for each time step. Finally, with an electricity cost of USD 0.126/kWh and the E_C, the aeration cost can be calculated using Equation (11). For anaerobic culture, the aeration cost is set as zero.

$$\$_{aeration} = 0.126\ E_C \tag{11}$$

Agitation in a bioreactor is used with the goal of keeping the homogeneity of the medium, and to help with the oxygen transfer rate. The agitation has to increase together with the increasing oxygen demand due to biomass formation throughout the culture in order to keep the oxygen concentration in the medium constant. Given that the oxygen concentration in the medium is being kept constant, the oxygen transfer rate is equal to the oxygen uptake rate, as shown in Equation (12).

$$OUR(t) = OTR(t) = k_L a(t)\ (C^*_{oxy} - C_{oxy,reac}) \tag{12}$$

Where $OTR(t)$ is the oxygen transfer rate at any given time (mmol/L.h), $k_La(t)$ is the volumetric mass transfer coefficient at any given time (h^{-1}), C^*_{oxy} is the oxygen concentration in the liquid phase at saturation for the medium conditions (0.21 mmol/L for 37°C), and $C_{oxy,reac}$ is the actual oxygen concentration in the medium (0.1 mmol/L). Rearranging Equation (12), $k_La(t)$ can be calculated. Now, the gas superficial velocity is given by Equation (13).

$$V_{super}(t) = \frac{Q_{air,out}(t)}{A} \tag{13}$$

Where $V_{super}(t)$ is the gas superficial velocity at any given time (m/s) and A is the area of the bioreactor, which in this case is 19.63 m^2. With the $k_La(t)$ and the $V_{super}(t)$ the Equation (14), a classic design equation as discussed by Humbird, Davis, and McMillan (2017), can be used.

$$k_La(t) = 0.002\left(\frac{P_S(t)}{V}\right)^{0.7} V_{super(t)}{}^{0.2} \tag{14}$$

Where here k_La is in s^{-1}, $P_S(t)$ is the gassed stirring power consumption (W) at any given time and V is the bioreactor volume (200 m^3). Rearranging Equation (14), $P_S(t)$ can be calculated. With the $P_S(t)$ (converted to kW), the energy consumed for stirring can be estimated using Equation (15).

$$E_S = \sum P_S\ \Delta t \tag{15}$$

Where E_S is the stirring total energy consumption (kWh) and when using MATLAB and DFBA, the Δt is the time step of the simulation and P_S the compressor power consumption for each time step. Finally. With an electricity cost of USD 0.126/kWh and the E_S, the agitation cost can be calculated using Equation (16).

$$\$_{agitation} = 0.126\ E_S \tag{16}$$

For the case of anaerobic culture, agitation is used mostly just to keep homogeneity, so no control system as a function of the $k_La(t)$ is needed, and the agitation can be less intense than the aerobic cultures. Therefore, adopting an agitation of 50 rpm (0.83 s^{-1}), the ungassed stirring power consumption can be estimated. First, the Reynolds number has to be calculated using Equation (17) (Hughmark, 1980; Junker, 2004; Michel & Miller, 1962; Van'T Riet, 1979; Yagi & Yoshida, 1975).

$$Re = \frac{\rho\ d^2 N}{\mu} \tag{17}$$

Where Re is the Reynolds number, ρ_{medium} is the medium density (1032 kg/m^3), d is the bioreactor diameter (5 m), N is the chosen agitation (0.83 s^{-1}) and assuming the same viscosity as water, μ is the medium viscosity (0.001 kg/s·m). This results in a Re of 4336335, categorized as turbulent flow. Using a flat-blade impeller (W/D = 1/5) and with the Re number of 4336335, a power number (c) of 4 can be found on Figure 1, and with Equation (18), the power required for ungassed mixing can be calculated (Hughmark, 1980; Junker, 2004; Michel & Miller, 1962; Van'T Riet, 1979; Yagi & Yoshida, 1975).

$$P_S = c\,\rho\,N^3 d^5 \tag{18}$$

The power required for ungassed mixing at 50 rpm and the bioreactor conditions is 136.1 kW. The stirring total energy consumption is then given by Equation (19).

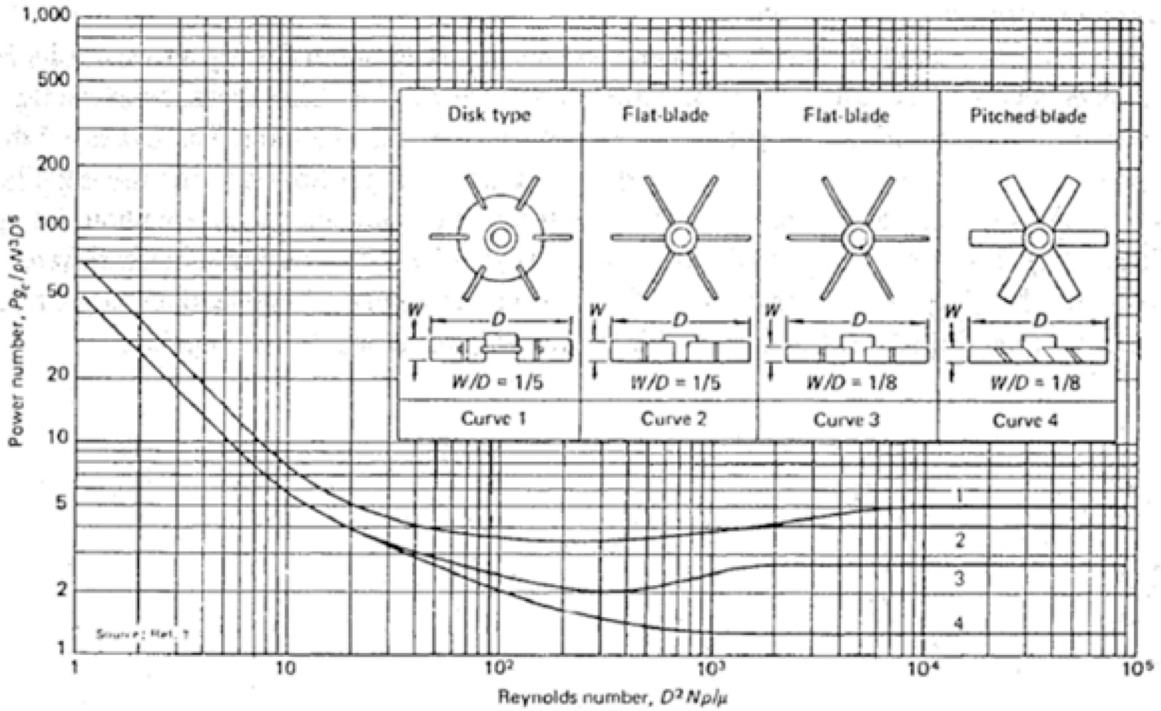

Figure 1: Power number curve for different types of impellers
Source: Bates, Fondy, and Corpstein (1963)

$$E_S = P_S \ t_{(op)} \tag{19}$$

Where $t_{(op)}$ is the operational time of the anaerobic batch simulated. Now the cost of agitation can be calculated with the previously established Equation (16).

Temperature control in a bioprocess is necessary because heat is released by the cell metabolism and the energy transferred from the impellers. The heat released by the cell can be related to the oxygen uptake rate according to Equation (20), in the case of an aerobic culture.

$$Q_{heat}(t) = K \ OUR(t) \ V \tag{20}$$

Where $Q_{heat}(t)$ is the metabolic heat release rate at any given time (kJ/h) and K is a constant of proportionality of 0.50 kJ/mmol O2. With $Q_{heat}(t)$ (converted to kW), the total metabolic energy generation can be calculated using Equation (21).

$$E_M = \sum Q_{heat} \ \Delta t \tag{21}$$

Where E_M is the total metabolic energy generation (kWh), and when using MATLAB and DFBA the Δt is the time step of the simulation and Q_{heat} is the metabolic heat release rate for each time step. Finally, with an electricity cost of USD 0.126/kWh, the E_M and the previously calculated E_S, the cooling costs can be calculated using Equation (22).

$$\$_{cooling} = 0.126 \ \frac{E_M + E_S}{n_b} \tag{22}$$

Where n_b is the efficiency number of thermostatic bath (0.7). For the case of anaerobic culture, rather than estimating the metabolic heat released as a function of the oxygen consumed, the heat generated can be related to the amount of glucose consumed. The work by Hannon, Bakker, Lynd, and Wyman (2007) gives a relation of 235 kJ per mol of glucose consumed. Therefore, the heat released due to metabolic activity in an anaerobic culture can be estimated using Equation (23).

$$E_M = \frac{K_{anae} \, S_{(cons)} \, V}{3600} \tag{23}$$

Where K_{anae} is the constant of proportionality of 235 kJ/mol glc and $S_{(cons)}$ is the total substrate consumed divided by the reactor volume (mol glc/L). With an electricity price tag of USD 0.126/kWh, the calculated E_M and the previously estimated E_S under anaerobic condition, the cooling costs for anaerobic culture can be calculated with the previously established Equation (22). The full MATLAB scripts to calculate the bioreactor operation costs after running an aerobic or anaerobic DFBA simulation during the growth or non-growth phase, in the conditions tested, is given in the Supplementary material D.

For the downstream costs, Gerngross (1999) summarizes the PHB production steps in Figure 2.

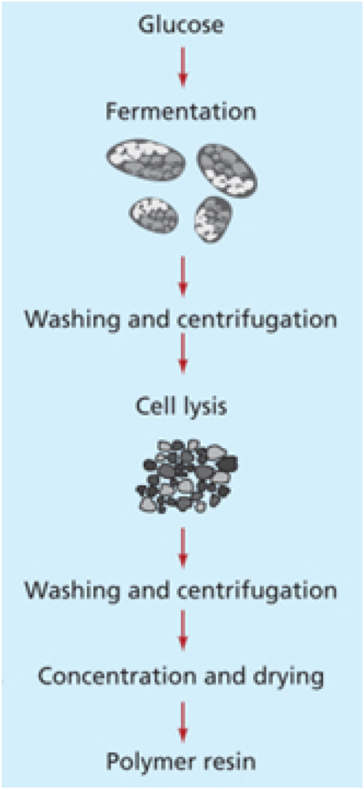

Figure 2: Simplified PHB production steps
Source: Gerngross (1999)

The works by Choi and Lee (1997), Gurieff and Lant (2007) and Harding et al. (2007) further details the downstream process as being compose of the following steps, homogenization, first centrifugation, extraction, washing and centrifugation, washing and centrifugation once again, drying, water and solid waste treatment. The homogenization process is estimated to consume 0.00035 kWh per gram biomass disrupted, so with the electricity price of USD 0.126/kWh and

the final biomass concentration, the cost with the homogenization step can be calculated for each simulation. The work by Szepessy and Thorwid (2018) points a centrifuge energy demand of 0.5 kWh per volume. So for the first centrifugation step in the process being simulated in this work, the energy demand for centrifugation of the 200 m^3 medium is 100 kWh, which implies a cost of USD 12.60 , given the energy price tag of USD 0.126/kWh. For the extraction step, the paper by Wu, Long, Zhang, Reed, and Maravelias (2018) presents a curve for estimating the cost of separation as a function of the product titer, as shown in Figure 3.

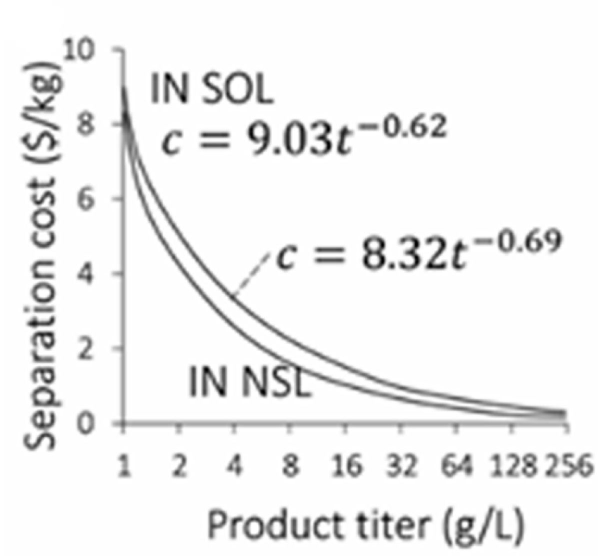

Figure 3: Separation cost as a function of the product titer
Source: Wu et al. (2018)

The intracellular non-soluble (IN NSL) in water curve can be applied to metabolites such as PHB. It can be seen that the separation cost drops exponentially with an increase in titer. As for the cost of solvent used in the extraction process, the paper by Pavan et al. (2019) points that PHB extraction requires 9.52 L of solvent/kg CDW, however, due to a solvent recovery system, a global solvent recovery of 99.82% can be achieved, so only 0.18% of the solvent used is actually new and has to be bought by the price of USD 1.15/L. The following washing and centrifugation step requires a total of four washes, using a value of 1/3 of the total medium volume for each wash, resulting in a total volume of 266.68 m^3 to be centrifuged, which in turn, has an energy demand of 133.34 kWh, costing then USD 16.8 (Harding et al., 2007). Then, the final washing and centrifugation only requires two washes, once again with a volume of 1/3 of the total medium each, so in this step a total of 133.34 m^3 has to be centrifuged in the process simulated in this work, leading to a cost of USD 8.4. For the drying step, according to Gerngross (1999), around 2kg of steam for each 1kg of PHB is required, so with the titer and the steam price tag of USD 0.004/kg, the cost of the drying step can be estimated. The last downstream step takes into consideration environment impacts in the form of water and solid waste treatment. The book Petrides (2013) provides a relation of around USD 0.5/m^3. In this work, the total wastewater volume is the sum of the culture medium and all the water used in the washing steps and the volume of solvent used on each simulation. The solid waste treatment cost is $0.05/kg CDW (Petrides, 2013).

Now, with the revenue from selling PHB and the costs associated with its production, the

gross profit per batch can be calculated. However, the profit per batch does not take into account the batch operation time, something that has to be considered because if the batch takes too long to reach a high titer, it may not be worth it. Much like the hypothesis pointed by Zhuang, Yang, Cluett, and Mahadevan (2013), the highest performance is not necessarily achieved with the highest yield, or the highest titer or highest productivity, but with the set of values of yield, titer, and productivity that when analyzed in relation to operational, upstream and downstream costs, leads to the highest gross profit. Precisely because of this, the metric adopted for the evaluation was the monthly gross profit. The operation time $t_{(op)}$ of a batch is the time required to reach the final titer obtained in that batch. The turnaround time $t_{(off)}$, which is the time needed for cleaning, preparation, and starting another batch was assumed to be 12 hours (Leong et al., 2017). The average month duration time was adopted as 30 days, equivalent to 720 hours. Thus, the number of batches that can be carried out in one month (N_{bat}) can be calculated according to Equation (24).

$$N_{bat} = \frac{720}{t_{(op)} + t_{(off)}} \tag{24}$$

If N_{bat} in Equation (24) is a decimal number, it needs to be rounded down, since the number of batches has to be a whole number and it is not possible to round up since it would not be possible to make that extra batch in the month. With the gross profit per batch, and the number of batches that can be done per month, the monthly gross profit can be calculated using Equation (25).

$$MGP = GP\, N_{bat} \tag{25}$$

Equation (25) allows estimating in a simplified way the gross profit obtained per month, as a function of parameters such as the biomass, yield, titer and the operation time (and with that, the productivity). This metric is ideal for evaluation of all the simulated cells with flux distributions that explore the trade-off between biomass and product formation and to evaluate possible two-phase PHB production approaches. By analyzing the monthly gross profit results obtained, the maximum theoretical monthly gross profit for each condition can be identified. Now, to calculate what is known as net profit, parameters such as employee salaries, fees, lease rent, maintenance costs, etc. would be required, but these parameters are no longer closely linked with the yield, titer and operation time. For the purpose of comparing performance between the simulated cells, whether using different microorganisms or operating in different conditions such as aerobiosis or anaerobiosis, estimating the monthly gross profit alone is already sufficient.

References

Bates, R. L., Fondy, P. L., & Corpstein, R. R. (1963). An examination of some geometric parameters of impeller power. *Industrial and Engineering Chemistry Process Design and Development*, *2*(4), 310–314. doi: 10.1021/i260008a011

Cardoso, V. M., Campani, G., Santos, M. P., Silva, G. G., Pires, M. C., Gonçalves, V. M., ... Zangirolami, T. C. (2020, jun). Cost analysis based on bioreactor cultivation conditions: Production of a soluble recombinant protein using Escherichia coli BL21(DE3). *Biotechnology Reports*, *26*, e00441. Retrieved from `https://linkinghub.elsevier.com/retrieve/pii/S2215017X1930637X` doi: 10.1016/j.btre.2020.e00441

Choi, J.-i., & Lee, S. Y. (1997). Process analysis and economic evaluation for Poly(3-hydroxybutyrate) production by fermentation. *Bioprocess Engineering*, *17*(6), 335.

Retrieved from http://link.springer.com/10.1007/s004490050394 doi: 10.1007/s004490050394

Clarke, K. G. (2013). *Bioprocess engineering*. Woodhead Publishing Limited. Retrieved from http://www.sciencedirect.com/science/book/9781782421672 doi: 10.1533/9781782421689

Dincer, I., & Zamfirescu, C. (2018). 1.5 Thermodynamic Aspects of Energy. In *Comprehensive energy systems* (Vol. 1-5, pp. 153–211). Elsevier. Retrieved from https://linkinghub.elsevier.com/retrieve/pii/B978012809597300105X doi: 10.1016/B978-0-12-809597-3.00105-X

Gerngross, T. U. (1999, jun). Can biotechnology move us toward a sustainable society? *Nature Biotechnology*, *17*(6), 541–544. Retrieved from http://www.nature.com/articles/nbt0699_541 doi: 10.1038/9843

Gurieff, N., & Lant, P. (2007, dec). Comparative life cycle assessment and financial analysis of mixed culture polyhydroxyalkanoate production. *Bioresource Technology*, *98*(17), 3393–3403. Retrieved from https://linkinghub.elsevier.com/retrieve/pii/S0960852407004610 doi: 10.1016/j.biortech.2006.10.046

Hanly, T. J., Urello, M., & Henson, M. A. (2012, mar). Dynamic flux balance modeling of S. cerevisiae and E. coli co-cultures for efficient consumption of glucose/xylose mixtures. *Applied Microbiology and Biotechnology*, *93*(6), 2529–2541. Retrieved from http://link.springer.com/10.1007/s00253-011-3628-1 doi: 10.1007/s00253-011-3628-1

Hannon, J. R., Bakker, A., Lynd, L. R., & Wyman, C. E. (2007). Comparing the scale-up of aerobic and anaerobic biological processes. *2007 AIChE Annual Meeting*.

Harding, K., Dennis, J., Vonblottnitz, H., & Harrison, S. (2007, may). Environmental analysis of plastic production processes: Comparing petroleum-based polypropylene and polyethylene with biologically-based poly-β-hydroxybutyric acid using life cycle analysis. *Journal of Biotechnology*, *130*(1), 57–66. Retrieved from https://linkinghub.elsevier.com/retrieve/pii/S0168165607001514 doi: 10.1016/j.jbiotec.2007.02.012

Hughmark, G. A. (1980). Power Requirements and Interfacial Area in Gas-Liquid Turbine Agitated Systems. *Industrial and Engineering Chemistry Process Design and Development*, *19*(4), 638–641. doi: 10.1021/i260076a023

Humbird, D., Davis, R., & McMillan, J. (2017, nov). Aeration costs in stirred-tank and bubble column bioreactors. *Biochemical Engineering Journal*, *127*, 161–166. Retrieved from http://dx.doi.org/10.1016/j.bej.2017.08.006https://linkinghub.elsevier.com/retrieve/pii/S1369703X17302103 doi: 10.1016/j.bej.2017.08.006

Junker, B. H. (2004, jan). Scale-up methodologies for Escherichia coli and yeast fermentation processes. *Journal of Bioscience and Bioengineering*, *97*(6), 347–364. Retrieved from https://linkinghub.elsevier.com/retrieve/pii/S1389172304702182 doi: 10.1016/S1389-1723(04)70218-2

Leong, Y. K., Show, P. L., Lan, J. C.-W., Loh, H.-S., Lam, H. L., & Ling, T. C. (2017, sep). Economic and environmental analysis of PHAs production process. *Clean Technologies and Environmental Policy*, *19*(7), 1941–1953. Retrieved from http://link.springer.com/10.1007/s10098-017-1377-2 doi: 10.1007/s10098-017-1377-2

Lopes, M., Belo, I., & Mota, M. (2014). Over-pressurized bioreactors: Application to microbial cell cultures. *Biotechnology Progress*, *30*(4), 767–775. doi: 10.1002/btpr.1917

Mediboyina, M. K., Holden, N. M., O'Neill, S., Routledge, K., Morrissey, B., Lawless, F., & Murphy, F. (2022). Upscale fermenter design for lactic acid production from cheese whey

permeate focusing on impeller selection and energy optimization. *Journal of Food Science and Technology*, *59*(6), 2263–2273. Retrieved from https://doi.org/10.1007/s13197-021-05239-6 doi: 10.1007/s13197-021-05239-6

MERCK. (2000). Microbiology Manual. *Unitech Communications, Faisalabad*, 237.

Michel, B. J., & Miller, S. A. (1962). Power requirements of gas-liquid agitated systems. *AIChE Journal*, *8*(2), 262–266. doi: 10.1002/aic.690080226

Pavan, F. A., Junqueira, T. L., Watanabe, M. D., Bonomi, A., Quines, L. K., Schmidell, W., & de Aragao, G. M. (2019, jun). Economic analysis of polyhydroxybutyrate production by Cupriavidus necator using different routes for product recovery. *Biochemical Engineering Journal*, *146*(November 2018), 97–104. Retrieved from https://doi.org/10.1016/j.bej.2019.03.009https://linkinghub.elsevier.com/retrieve/pii/S1369703X19300889 doi: 10.1016/j.bej.2019.03.009

Petrides, D. (2013). *Bioprocess Design and Economics*.

Ramsay, B. A., Lomaliza, K., Chavarie, C., Dubé, B., Bataille, P., & Ramsay, J. A. (1990, jul). Production of poly-(beta-hydroxybutyric-co-beta-hydroxyvaleric) acids. *Applied and Environmental Microbiology*, *56*(7), 2093–2098. Retrieved from http://www.ncbi.nlm.nih.gov/pubmed/2117877http://www.pubmedcentral.nih.gov/articlerender.fcgi?artid=PMC184565https://journals.asm.org/doi/10.1128/aem.56.7.2093-2098.1990 doi: 10.1128/aem.56.7.2093-2098.1990

SPIRAX SARCO. (2021). *STEAM ENGINEERING PRINCIPLES AND HEAT TRANSFER.* Retrieved 2021-10-01, from https://www.spiraxsarco.com/learn-about-steam/steam-engineering-principles-and-heat-transfer/methods-of-estimating-steam-consumption?fbclid=IwAR0OB_wsbFyxRM48A7GE2k5GUffj7U6Dm71iVSgMpNeVn2PaJp_Nl-lpJzg#article-top

Szepessy, S., & Thorwid, P. (2018). Low Energy Consumption of High-Speed Centrifuges. *Chemical Engineering and Technology*, *41*(12), 2375–2384. doi: 10.1002/ceat.201800292

Van Ness, H. C., Smith, J. M., & Abbott, M. M. (2000). *Introdução à Termodinâmica da Engenharia Química*.

Van'T Riet, K. (1979). Review of Measuring Methods and Results in Nonviscous Gas-Liquid Mass Transfer in Stirred Vessels. *Industrial and Engineering Chemistry Process Design and Development*, *18*(3), 357–364. doi: 10.1021/i260071a001

Wu, W., Long, M. R., Zhang, X., Reed, J. L., & Maravelias, C. T. (2018, sep). A framework for the identification of promising bio-based chemicals. *Biotechnology and Bioengineering*, *115*(9), 2328–2340. Retrieved from https://onlinelibrary.wiley.com/doi/10.1002/bit.26779 doi: 10.1002/bit.26779

Yagi, H., & Yoshida, F. (1975). Gas Absorption by Newtonian and Non-Newtonian Fluids in Sparged Agitated Vessels. *Industrial and Engineering Chemistry Process Design and Development*, *14*(4), 488–493. doi: 10.1021/i260056a024

Zhuang, K., Yang, L., Cluett, W. R., & Mahadevan, R. (2013, dec). Dynamic strain scanning optimization: an efficient strain design strategy for balanced yield, titer, and productivity. DySScO strategy for strain design. *BMC Biotechnology*, *13*(1), 8. Retrieved from BMCBiotechnologyhttps://bmcbiotechnol.biomedcentral.com/articles/10.1186/1472-6750-13-8 doi: 10.1186/1472-6750-13-8
