# Supplementary figures and images for: Analysis of Poly-3-Hydroxybutyrate Production with Different Microorganisms Using the Dynamic Simulations for Evaluation of Economic Potential Approach
Source: ACS Omega. 2025 Jun 11;10(26):27756–74. doi: 10.1021/acsomega.4c11178 (PMC12242656; doi:10.1021/acsomega.4c11178)

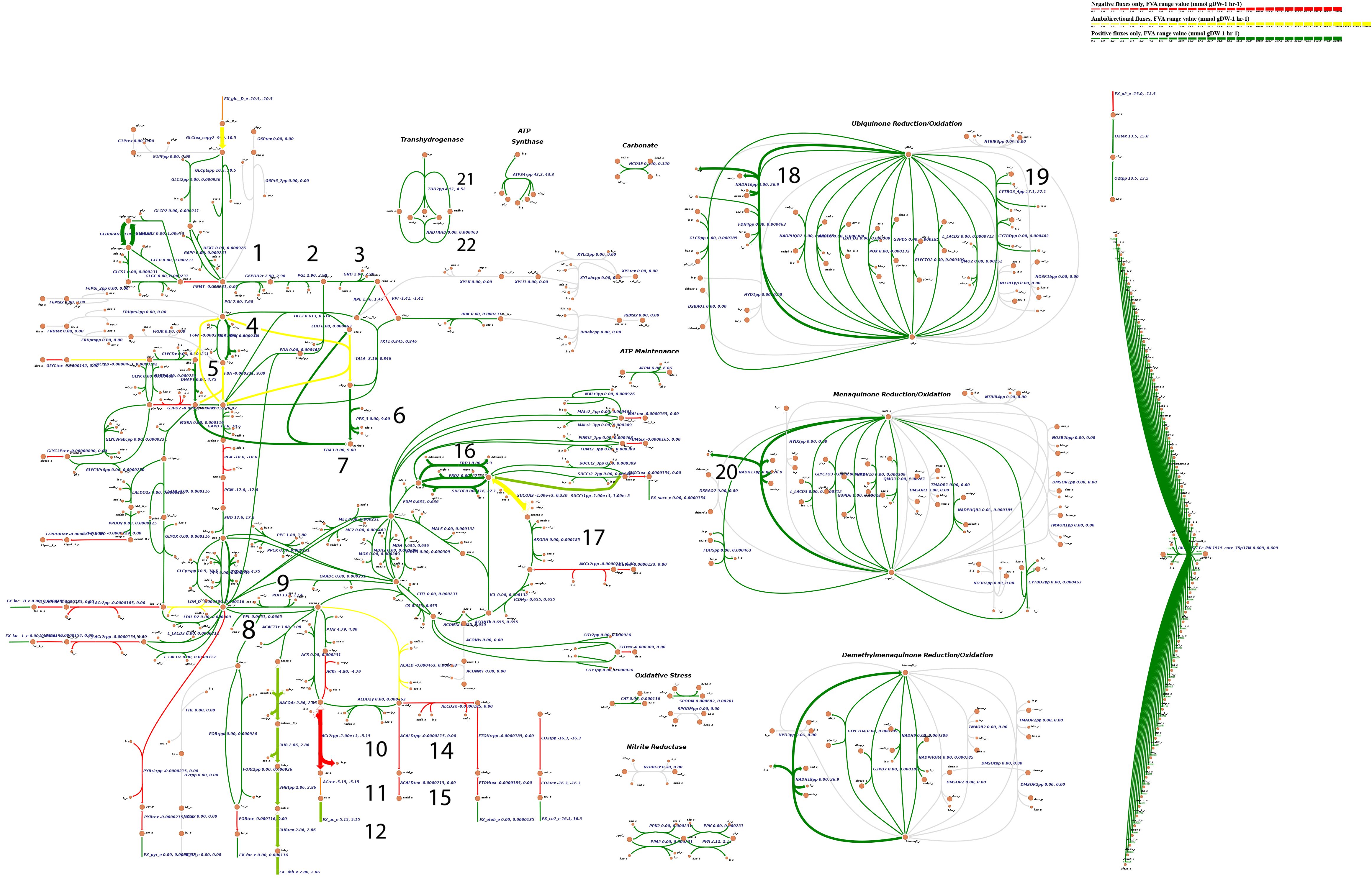

Supplement: Supplementary file 1 [file ao4c11178_si_001.zip › Supporting Information/Supporting Information G/E. coli constrained with experimental data from literature/experimental aerobic with numbers.png]
